# Wireless soft millirobots for climbing three-dimensional surfaces in confined spaces

**DOI:** 10.1126/sciadv.abn3431

**Published:** 2022-05-27

**Authors:** Yingdan Wu, Xiaoguang Dong, Jae-kang Kim, Chunxiang Wang, Metin Sitti

**Affiliations:** 1 Physical Intelligence Department, Max Planck Institute for Intelligent Systems, Stuttgart, 70569, Germany.; 2 Department of Mechanical Engineering, Vanderbilt University, Nashville, TN 37235, USA.; 3 Institute for Biomedical Engineering, ETH Zürich, Zürich 8092, Switzerland.; 4 School of Medicine and College of Engineering, Koç University, Istanbul 34450, Turkey.

## Abstract

Wireless soft-bodied robots at the millimeter scale allow traversing very confined unstructured terrains with minimal invasion and safely interacting with the surrounding environment. However, existing untethered soft millirobots still lack the ability of climbing, reversible controlled surface adhesion, and long-term retention on unstructured three-dimensional (3D) surfaces, limiting their use in biomedical and environmental applications. Here, we report a fundamental peeling-and-loading mechanism to allow untethered soft-bodied robots to climb 3D surfaces by using both the soft-body deformation and whole-body motion of the robot under external magnetic fields. This generic mechanism is implemented with different adhesive robot footpad designs, allowing vertical and inverted surface climbing on diverse 3D surfaces with complex geometries and different surface properties. With the unique robot footpad designs that integrate microstructured adhesives and tough bioadhesives, the soft climbing robot could achieve controllable adhesion and friction to climb 3D soft and wet surfaces including porcine tissues, which paves the way for future environmental inspection and minimally invasive medicine applications.

## Teaser

Wireless millimeter-scale soft climbing robots can traverse complex 3D tissue surfaces in enclosed and confined spaces.

## INTRODUCTION

Wireless miniature mobile robots promise to revolutionize environment inspection (*1*, *2*) and biomedical engineering (*3*, *4*) by enabling access to unstructured enclosed and confined spaces with agile maneuverability. Compared with their rigid-body counterparts, soft-bodied miniature robots could realize versatile locomotion by programming their diverse soft-body deformation modes (*5*, *6*) and safely interacting with their surrounding environment. Exciting advances have been made in recent years on soft miniature robots that are capable of traversing complex terrains using bioinspired locomotion modes, such as rolling (*7*), crawling (*8*), and swimming (*7*, *9*, *10*). Untethered soft miniature robots could also realize cargo delivery and manipulation functions for potential applications, such as biofluid pumping (*11*) and drug delivery (*12*–*14*). However, existing wireless soft miniature robots still lack the ability of climbing, reversible controlled surface adhesion, and long-term retention (*15*) on complex and unstructured three-dimensional (3D) surfaces, limiting their operation space and maneuverability. Once equipped with the ability to climb complex 3D surfaces in confined and enclosed spaces, they could reach previously inaccessible locations for environmental monitoring and exploration by distributing miniaturized wireless electronic devices (*16*) and even reach the disease sites inside the human body and perform minimally invasive biomedical functions, which are impossible or extremely challenging for other locomotion modes (*7*).

So far, untethered miniature climbing soft robots that are capable of inverted and vertical climbing on diverse 3D surfaces have not been achieved yet. The controllable (e.g., switchable) adhesion mechanisms in existing climbing robots include electroadhesion (*17*, *18*), van der Waals forces (*19*, *20*), and other dry adhesives (*21*), which only work for specific dry surfaces, limiting their applications in unstructured environments. The ability to climb wet surfaces is more challenging but would enable many critical applications. Climbing robots based on a vacuum suction mechanism at the centimeter scale (*22*) are tethered, requiring bulky pneumatic actuation units, and could only climb rigid wet surfaces, excluding soft surfaces. Recently developed electrically controlled adhesive hydrogels (*23*) could allow robots to adhere to wet surfaces, but they typically require conductive surfaces for switchable adhesion. Therefore, to achieve the climbing of untethered millimeter-scale soft robots on diverse 3D surfaces including wet and soft surfaces, a more generic controllable (e.g., switchable) adhesion mechanism has to be developed.

**Fig. 1.**
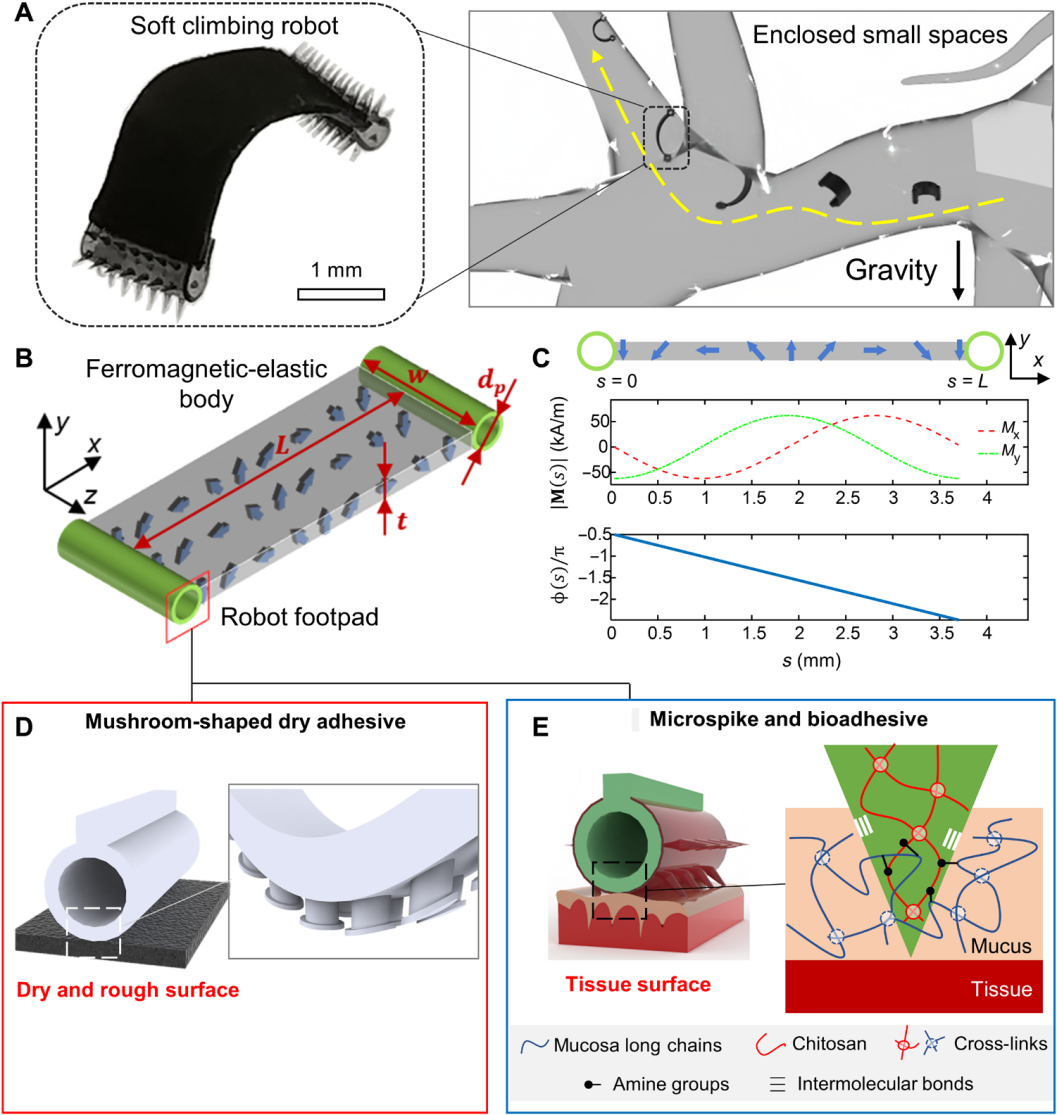
Wireless millimeter-scale soft climbing robot capable of traversing complex 3D surfaces in enclosed and confined spaces. (**A**) The concept of the soft climbing robot reaching spots previously inaccessible in enclosed small spaces. Left: Image of the deformed soft climbing robot controlled by external magnetic fields. Right: Illustration of climbing in enclosed small spaces. (**B**) Dimensions of the soft climbing robot with a ferromagnetic soft body (length, *L* = 3.7 mm; width, *w* = 1.5 mm; thickness, *t* = 150 μm). The robot has two nonmagnetic soft footpads with a flat surface for rigid substrates or a structured surface for slippery and deformable surfaces. (**C**) The magnetization profile of the soft climbing robot. Magnitude profile, *M*(*s*); phase profile, ϕ(*s*), *s* ∈ [0, *L*]. The blue arrows indicate the distribution of magnetic dipole moments. (**D**) Illustration of the robot footpad with mushroom-shaped dry adhesives for climbing 3D dry and rough surfaces. (**E**) Illustration of the unique robot footpad with both coated bioadhesives and microspikes for climbing tissue surfaces covered by mucus. Photo credit: Yingdan Wu and Xiaoguang Dong, Max Planck Institute for Intelligent Systems.

Here, we propose a fundamental peeling-and-loading mechanism compatible with various dry and wet adhesive footpads to create untethered soft-bodied robots that can climb diverse 3D dry and wet surfaces in confined spaces (Fig. 1A). Our generic peeling-and-loading mechanism enables the adhesive footpad attachment and detachment by using both the soft-body deformation and whole-body motion of the robot, controlled by external magnetic fields. This mechanism is implemented using different adhesive footpad designs, allowing the soft robot to achieve vertical and inverted climbing on diverse 3D surfaces with complex geometries, different surface roughness and softness, and even on soft tissue surfaces. With a unique robot footpad design that integrates both microstructures and tough bioadhesives, the soft climbing robot is demonstrated to achieve controllable adhesion and friction to locomote on various porcine tissue surfaces ex vivo. Our main contribution here is the peeling-and-loading mechanism and the unique robot footpad design, which enable untethered soft climbing robots on various wet and soft surfaces. We use external magnetic fields as the actuation method as reported in several works on magnetically actuated soft robots (*7*–*10*), as it allows a smaller robot size, wireless operation, and precise and fast control of the robot deformation and motion in contrast to other on-board actuation methods for climbing robots (*17*, *18*). To the best of our knowledge, there is still no untethered soft climbing robot at the millimeter scale because of the current limitations in miniaturization of on-board actuators and power sources.

With the climbing capability enabled by the peeling-and-loading mechanism and the unique robot footpad design, the robot is further integrated with various functions. First, we demonstrate that the robot could carry cargos with a large weight and volume, such as soft capsules for liquid drug delivery and miniaturized wireless electronic sensors for environment monitoring in the future. The robot still retains the previously reported soft-bodied multimodal locomotion capability (*7*), such as rolling, crawling, and swimming, to traverse other unstructured terrains in confined environments. Second, we demonstrate that the soft climbing robot could overcome the mucus layer barrier (*24*) with the microstructures on its footpads, stay for a relatively long time (hours) against external disturbances thanks to the adhesive footpads and the weak-adhesive body design, and release the microparticles loaded in the footpad microstructures by a swelling mechanism at a targeted location triggered by a specific pH threshold value. With the unique ability to climb diverse complex 3D surfaces, our proposed soft climbing robot could pave the way for a wide range of critical applications in environmental inspection by accessing enclosed small spaces to distribute miniaturized wireless electronic sensors and in minimally invasive medicine by accessing unprecedented areas for biopsy and other medical functions.

## RESULTS

### Concept and design of the soft climbing robot

We present a soft climbing robot consisting of a ferromagnetic-elastic sheet–shaped body (length, *L* = 3.7 mm; width, *w* = 1.5 mm; and thickness, *t* = 150 μm) for soft-body deformation and whole-body motion, and two soft cylindrical ring–shaped footpads (diameter, *d_p_* = 500 μm) for controllable friction and adhesion as shown in Fig. 1B. Please note that the soft-body deformation is the shape-morphing behavior of the robot body, while the whole-body motion is the rigid-body translation and rotation of the robot. The robot body is designed with a sinusoidal magnetization profile **M**(*s*) along its length direction [magnitude profile, *M*(*s*); phase profile, ϕ (*s*), *s* ∈ (0, *L*)] as shown in Fig. 1C. We design the robot body with such a magnetization profile because of three reasons. First, the magnetization profile allows a desired large deformation upon applying a uniform magnetic field so that the robot could increase and decrease the distance between its two footpads sequentially to move forward with a relatively large step size. Second, this magnetization profile allows a relatively large magnetic moment and therein a large whole-body torque so that the robot footpad could be peeled off from and loaded onto the substrate surfaces in the climbing locomotion mode. Last, the magnetization profile could also enable other desired time-varying shapes to achieve other locomotion modes as reported previously in (*7*).

To achieve the climbing locomotion, the soft robot needs a symmetry-breaking body deformation to move forward and a mechanism to allow switchable attachment and detachment, as well as sufficient friction when contacting the 3D surfaces. The first key component for climbing is the peeling-loading mechanism (see section “*Mechanism of controlled peeling-and-loading and soft-body deformation for climbing*”) by controlling the whole-body rotation to switch the directions of the boundary forces for rapid attachment and detachment from the robot footpads to the surface. As the second key component for climbing, we can control the robot soft-body deformation by controlling the applied distributed magnetic torque with a precisely applied external magnetic field (see section “*Mechanism of controlled peeling-and-loading and soft-body deformation for climbing*”). The soft-body deformation of the robot allows varying the distance between the two footpads to produce whole-body motion. The last key component for climbing is a unique adhesive footpad design, as illustrated in Fig. 1 (D and E). The footpad design with proper dry adhesives allows climbing dry and rough surfaces (Fig. 1D). To climb wet and soft tissue surfaces, the footpad design uses both microspikes for enhanced friction and the chitosan-based bioadhesive for enhanced adhesion on mucus-covered tissue surfaces (Fig. 1E). The microspikes provide the pad with enhanced friction, as they can penetrate the mucus layer. At the same time, the chitosan-based bioadhesive (*25*, *26*) offers a strong attachment of the footpads to the mucus layer. Note that our robot is able to climb different dry and wet surfaces as long as the proper dry and wet adhesives are integrated into the ring-shaped robot footpads. Although adhesives such as dry adhesives and bioadhesives have been reported before, it is nontrivial to integrate them to develop an untethered soft climbing robot at the millimeter scale, which requires fundamental design of the robot footpad structure and intelligent control of the robot body deformation and motion to enable climbing 3D, wet, and soft surfaces.

### Mechanisms of controllable peeling-and-loading and soft-body deformation for climbing

**Fig. 2.**
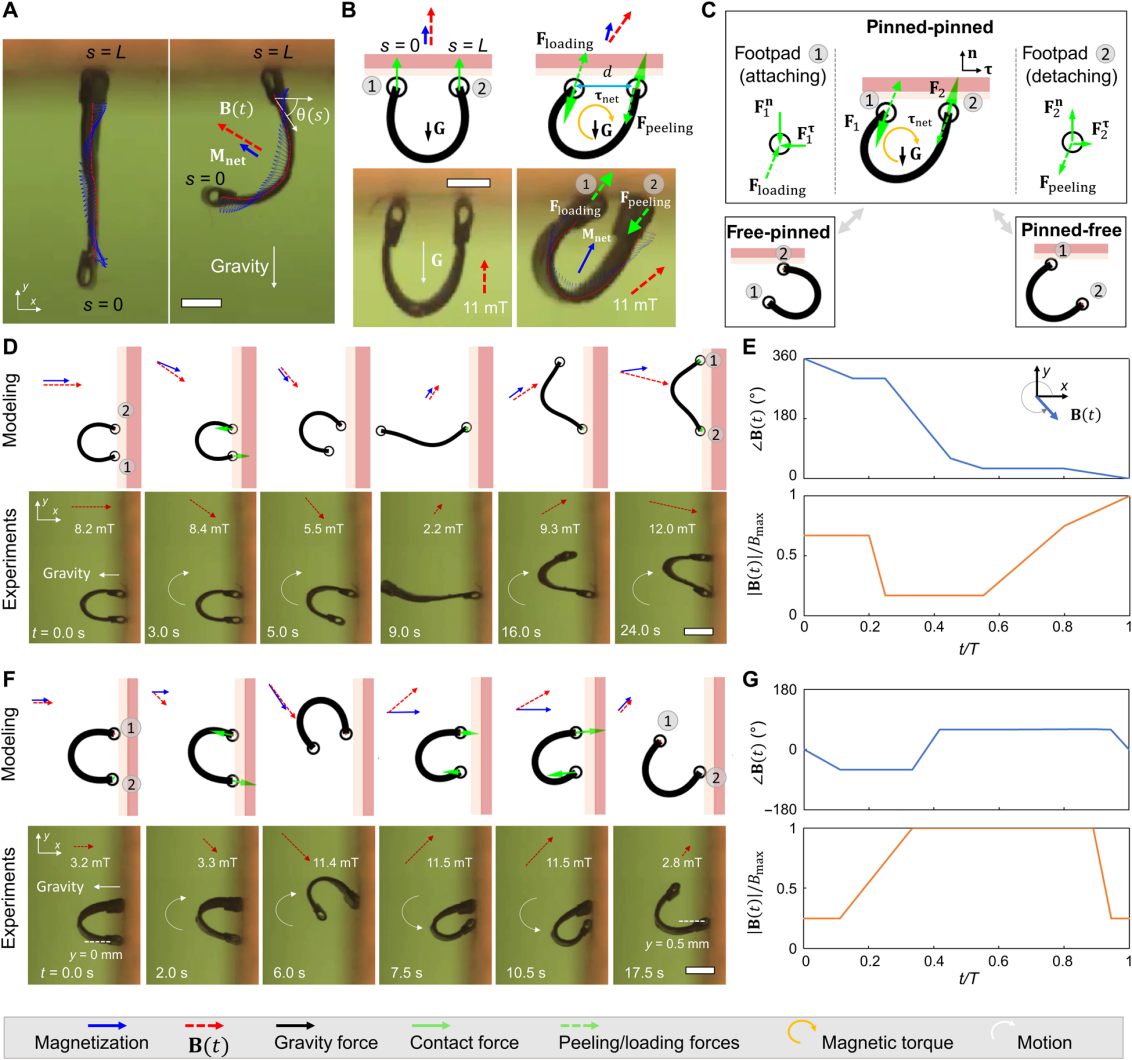
Soft robot climbing locomotion gaits enabled by the controllable peeling-and-loading mechanism. (**A**) Images of the soft robot during inverted climbing and its overlapped magnetization profiles after deformation. ∣**B**∣ = 11 mT. (**B**) The schematics and experimental images of the peeling and loading mechanism when the robot adheres to a surface. The whole-body torque **τ**_net_ (yellow curved arrows) imposed by an external magnetic field induced the loading and peeling forces (green arrows) from the robot footpads to the substrate. (**C**) Illustration of three types of boundary conditions for the soft climbing robot. **F**_1_ and **F**_2_ denote the contact forces applied from the substrate surface to the robot footpads “1” and “2.” The superscripts “**n**” and “**τ**” indicate the normal and shear components, respectively. (**D**) The tumbling-based climbing gait. Top row: The modeled tumbling-based climbing gait with the corresponding magnetic fields. Bottom row: Video snapshots (movie S1) of the robot climbing a solid plywood substrate surface. (**E**) The corresponding applied magnetic field waveform in the tumbling-based climbing gait in (D). (**F**) The walking-based climbing gait. Top row: The modeled walking-based climbing gait together with the corresponding magnetic fields. Bottom row: Video snapshots (movie S1) of the robot climbing a solid plywood substrate surface. (**G**) The corresponding applied magnetic fields in the walking-based climbing gait in (F). Photo credit: Yingdan Wu and Xiaoguang Dong, Max Planck Institute for Intelligent Systems.

In Fig. 2 and movie S1, we present the mechanism of controlling the soft-body deformation and the peeling-and-loading behavior of the robot footpads, as well as the corresponding magnetic field waveforms. Upon applying an external magnetic field **B**(*t*), the robot body will deform into a specific shape parameterized with the deflection angle θ(*s*) subject to a distributed magnetic torque **τ**(*s*) when one footpad is adhering to the surface (Fig. 2A). Moreover, to achieve the loading and peeling behaviors, Fig. 2B shows that the deformed robot body also experiences a nonzero net magnetic torque **τ**_net_ = **M**_net_ × **B**(*t*) (yellow curved arrows), where **M**_net_ is the net magnetic moment given by $M_{\text{net}}=\int_{0}^{L}R[\theta (s)]M(s)\;\mathrm{wt}ds$, where **R** denotes the rotation matrix. **τ**_net_ can be controlled to be either clockwise (CW) or counterclockwise (CCW) by varying the rotating direction of **B**(*t*), such that pad 1 (*s* = 0) is loaded, while pad 2 (*s* = *L*) is peeled off, or vice versa. The peeling or loading force could be approximately estimated by ∣**F**_peeling,n_∣ = ∣**F**_loading,n_∣ = ∣**τ**_net_∣/*d* based on a whole-body moment balance equation (see Supplementary note S1), where the subscript n indicates the force in the surface normal direction, assuming that the robot is in an equilibrium state, and its gravity force is negligible compared with the peeling and loading forces.

We use a simplified solid-mechanics model (*6*) based on the Euler-Bernoulli beam theory to help us understand the soft-body deformation and the peeling-and-loading mechanism by controlling external magnetic fields. As shown in Fig. 2C, with the peeling-and-loading mechanism, the climbing locomotion of our robot further involves three types of boundary conditions in a complete locomotion cycle, including “pinned-free,” “pinned-pinned,” and “free-pinned.” First, the pinned-free boundary condition means that pad 1 is adhering to the substrate with only one rotational degree of freedom (DOF), while pad 2 is free to move. Second, pinned-pinned means that both pads are adhering to the substrate so that they cannot move. Third, free-pinned indicates that pad 2 is adhering to the substrate with only one rotational DOF, while pad 1 is free to move. By sequentially transiting between the three boundary conditions and concurrently controlling the distance between its two footpads in the pinned-free or free-pinned conditions, the robot could achieve the climbing locomotion.

Figure 2 (D and F) show two possible climbing locomotion gaits of our robot: tumbling- and walking-based climbing gaits. In the tumbling-based climbing gait (Fig. 2, D and E), with the pinned-free boundary condition, the robot body deforms gradually and reaches a “C shape” when increasing the magnitude of **B**(*t*) up to about 8 mT. When further rotating **B**(*t*) in the CW direction, the whole-body torque induced by the nonzero net magnetic torque **τ**_net_ turns the robot body toward the substrate surface to load pad 1. Once both footpads make contact with the substrate, the boundary condition switches to the pinned-pinned configuration, where further rotating **B**(*t*) increases **τ**_net_ to load pad 2 onto the substrate, while pad 1 is peeled off. **B**(*t*) is then decreased to relax the robot body and then increased to about 12 mT in the opposite direction to deform the robot into a “V shape.” Further rotating **B**(*t*) in the CW direction produces the whole-body torque to load pad 1 and peel off pad 2 from the substrate surface. In this gait, pad 1 and pad 2 repeatedly become the front pad when the robot body flips over. The tumbling gait has the maximum allowable peeling and loading torques when **B**(*t*) is perpendicular to **M**_net_, which allows strong preload and pull-off forces for attaching and detaching, respectively.

Similarly, the walking-based climbing gait (Fig. 2, F and G) also involves the peeling and loading processes enabled by **τ**_net_, while the robot body motion is realized by repeatedly increasing and decreasing the robot footpad distances by the soft-body deformation. With pad 1 pinned, the robot body changes from a wide C shape to a narrow C shape when increasing the magnitude of **B**(*t*) (~ 11.5 mT), allowing pad 2 to move forward. In contrast, with pad 2 pinned, decreasing the magnitude of **B**(*t*) (~ 3.3 mT) allows the robot body to change from a narrow C shape to a wide C shape so that pad 2 moves forward. Compared with the tumbling-based climbing gait, the walking-based climbing gait results in smaller peeling and loading forces due to smaller net magnetic torques but allows a finer step size.

### Robot footpad design and locomotion control for climbing 3D dry and rough surfaces

**Fig. 3.**
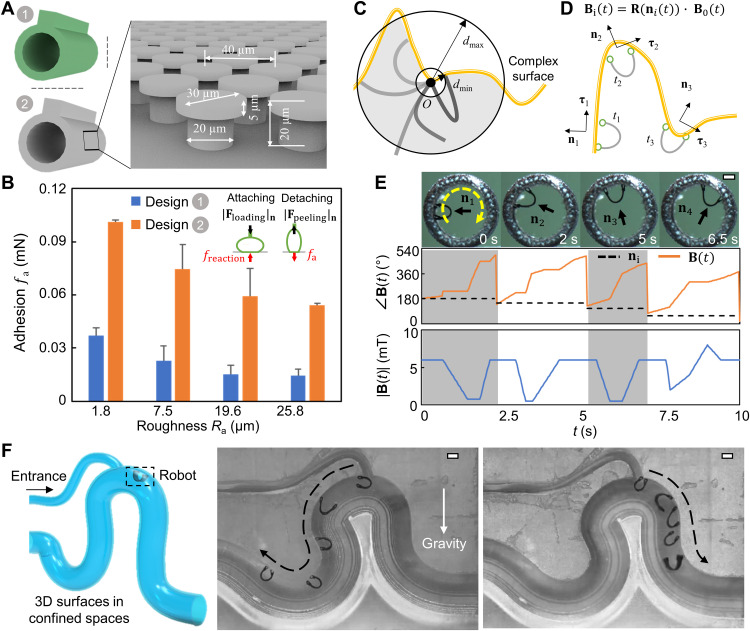
Robot footpad design and locomotion control for climbing 3D dry surfaces. (**A**) Schematics of two robot footpad designs. Design 1, robot footpad without any dry adhesive. Design 2, robot footpad with patterned mushroom–shaped gecko-inspired adhesive. (**B**) The adhesion measurement of two robot footpads on solid substrates with different roughness under preload of 0.1 mN. (**C**) Schematics of the reachable region (gray colored area) of the free end of the robot on a curved surface (yellow curve). *O* represents the anchoring point where the robot adheres and pins to the surface. (**D**) Illustration of parameterizing a curved surface for planning the robot climbing locomotion. **n**_i_ and **τ**_i_ denote the local surface normal and tangential directions, respectively. The magnetic field is adjusted accordingly by **B**_i_(*t*) = **R**[**n**_i_(*t*)]∙ **B**_0_(*t*), where **R**[**n**_i_(*t*)] is the rotation matrix and **B**_0_(*t*) denotes the original magnetic field given in Fig. 2 (E and G). (**E**) Video snapshots (top row; movie S2) and example magnetic field angle (bottom row) of the robot climbing the inner surface of a grinding paper roll. (**F**) Video snapshots (movie S2) of the soft climbing robot during inverted and vertical climbing on a phantom model with enclosed and confined spaces. In all figures, scale bars, 1 mm. Photo credit: Yingdan Wu and Xiaoguang Dong, Max Planck Institute for Intelligent Systems.

Figure 3 and movie S2 show that the robot footpads could be designed on demand to allow the robot to climb 3D dry surfaces with different roughness, while the control signal could be adapted to allow the robot to climb complex curved surfaces. We use mushroom-shaped dry adhesives (*27*) for dry surfaces as the adhesion could be widely tuned by designing the mushroom-shaped fibril structures (*28*–*31*). We compare the robot footpad without any adhesive (design 1) and that with mushroom-shaped dry adhesives (design 2) as illustrated in Fig. 3A. When varying the roughness of the tested surfaces, design 2 shows a wider range of tunable adhesion under different preloads (Fig. 3B). For a surface with an average roughness of 25.8 μm, design 2 still maintains an adhesion of 0.054 mN and 0.264 mN with a preload force of 0.1 mN and 0.5 mN, respectively, while design 1 shows an adhesion less than the robot gravity (0.03 mN) with even a preload of 0.5 mN. This is because the fibril structures can conform better to the local asperities (*32*) in design 2, resulting in higher adaptability to rough surfaces.

With a unique robot footpad design, the peeling-and-loading mechanism, and the soft-body deformation, we show that our soft climbing robot could achieve vertical and inverted climbing on 3D dry and rough surfaces with a wide range of reachability and maneuverability. First, Fig. 3C illustrates the reachability of the robot for surfaces with a complex boundary curvature profile. When the robot is at a pinned-free boundary condition where one pad is pinned at a point *O*, the effective reachable space of the free pad is defined as the intersection between the ring-shaped area with inner and outer diameters of *d*_min_ and *d*_max_ and the free space bounded by the surface profile. Here, *d*_min_ and *d*_max_ are the minimum and maximum values of the distance *d* between the two pads, respectively. Note that *d*_max_ is less than the robot body length *L* because the robot body must deform to obtain a sufficient large **M**_net_ and therein a large **τ**_net_ such that the peeling and loading forces could be produced effectively. In addition to the body geometric and material parameters, *d*_min_ depends on the maximum achievable **B**(*t*) in a magnetic actuation system. By planning the motion steps of the robot in each locomotion cycle, the robot can climb complex surfaces with a wide range of reachability.

Second, Fig. 3D illustrates that the robot could also climb surfaces with large curvatures, either convex or concave. The desired **B**(*t*) could be obtained by rotating the baseline magnetic field **B**_0_(*t*) (Fig. 2, E and G) for climbing a flat surface according to a rotation matrix **R**. Here, **B**(*t*) is given by **B**(*t*) = **R**(**n**(*x*, *y*, *z*))**B**_0_(*t*), where **n**(*x*, *y*, *z*) denotes the surface normal vector at a specific location. The robot thus could be controlled to adapt to various curved surfaces. For example, Fig. 3E illustrates how **B**(*t*) is adjusted to enable the robot to climb a curved 3D rough surface. In this example, the robot climbs the curved surface at selected waypoints with different surface normal vectors in the *x*-*z* plane. By rotating **B**_0_(*t*) according to the direction of the surface normal vector, the resulting magnetic waveform **B**(*t*) allows the robot to climb the highly curved surfaces.

Last, in Fig. 3F, we demonstrate that our robot could have vertical and inverted climbing in torturous and confined tubular structures with complex geometry. With external magnetic actuation, the soft robot could be wirelessly controlled from the outside to climb in very confined and enclosed spaces such as torturous pipes to deliver miniaturized wireless electronic sensors (*16*) to monitor and explore hazardous environments (*33*).

### Unique robot footpad design for controllable adhesion and friction on 3D soft tissue surfaces

In addition to 3D dry surfaces, we use the control mechanisms of the soft robot for climbing soft tissues. Compared with solid and dry substrate surfaces, tissue surfaces vary considerably in terms of not only the geometry but also the softness and thickness of the mucus layer, presenting substantial challenges for the climbing locomotion in terms of adhesion control (*34*). For example, the bronchi tissues in the respiratory tract consist of a relatively hard back layer called cartilage with an elastic modulus up to 6.7 MPa and a soft inner layer (elastic modulus, 28.4 kPa) made of smooth muscles covered by a 70-μm-thick mucus layer. In comparison, the stomach tissue is a soft tissue (elastic modulus, 1.9 kPa) with a mucus layer as thick as 300 μm (*35*–*38*).

**Fig. 4.**
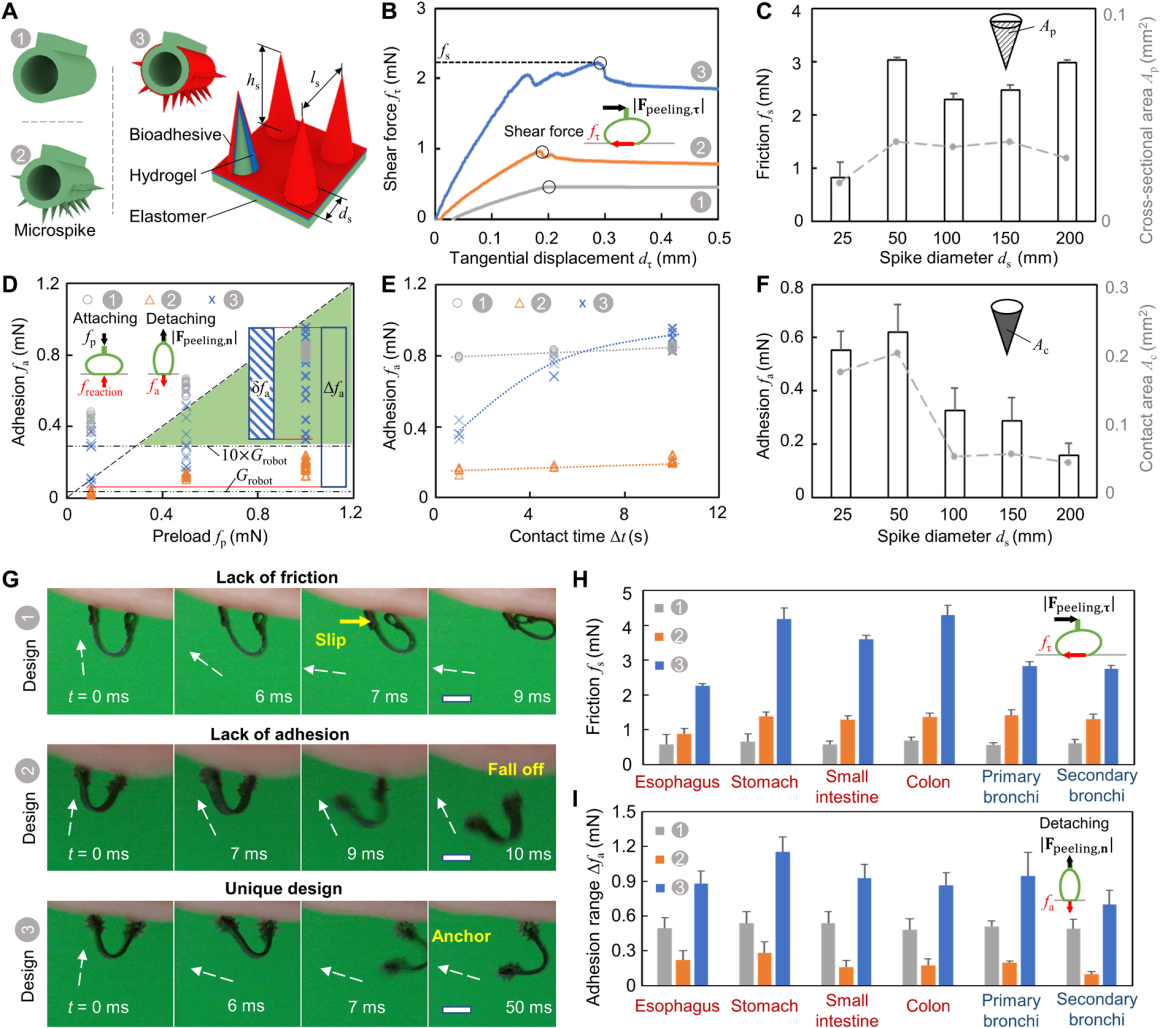
Design of the robot footpad microstructure and adhesive for controllable adhesion and friction. (**A**) Three representative robot footpad designs. Design 1, plain footpad; design 2, footpad with microspikes; design 3, footpad with microspikes coated by bioadhesives. Microspikes: diameter, *d*_s_ = 100 μm; height, *h*_s_ = 200 μm; spacing *l*_s_ = 400 μm. (**B**) The shear force *f*_τ_ as a function of tangential displacements. The footpad friction *f*_s_ is equal to the maximum *f*_τ_ before slipping. (**C**) *f*_s_ (left axis) and vertical cross area *A*_p_ (right axis) in design 3 as a function of the spike diameter *d*_s_. *h*_s_/*d*_s_ = 2 and *l*_s_/*h*_s_ = 1. *A*_p_ = 0.5 ∙ *n*_s_*h*_s_*d*_s_. *n*_s_: spike number per row. (**D**) The pad adhesion *f*_a_ as a function of the preload *f*_p_. Contact time Δ*t* =1, 5, 10 s. Total adhesion range, Δ*f*_a_ = max *f*_a_ – min *f*_a_; adhesion range with a constant preload, δ*f*_a_. The green area denotes the desired adhesion-preload property. *G*_robot_: robot gravity force. (**E**) *f*_a_ as a function of the contact time Δ*t* when *f*_p_ = 1 mN. (**F**) The adhesion of design 3 as a function of the spike diameter *d*_s_. *h*_s_/*d*_s_ = 2; *l*_s_/*h*_s_ = 1. The contact area of the spikes: *A*_c_ = *n*_s_π*h*_s_*d*_s_. An additional 0.15 mm^2^ is added for the footpad with *d*_s_ ≤ 50 μm. (**G**). Video snapshots (movie S3) of the interacting behaviors between three footpad designs and the tissue surfaces. **B** field (white arrows), 12 mT. Scale bars, 1 mm. (**H** and **I**) *f*_s_ (H) and (I) Δ*f*_a_ of the three footpad designs on different porcine tissue surfaces. Tissues used in (B) to (G) are from a fresh porcine esophagus. In all figures, the error bar represents the SD for *n* = 5 measurements. Photo credit: Yingdan Wu and Xiaoguang Dong, Max Planck Institute for Intelligent Systems.

To further achieve the climbing locomotion on targeted tissue surfaces, the robot footpads are designed to be equipped with both microstructures and bioadhesives for a desired range of adhesion and friction, which are tunable by varying **B**(*t*). In Fig. 4, we justify the effectiveness of integrating both microstructures and bioadhesives in the robot footpads by comparing the friction and adhesion provided by three types of robot footpad designs. As shown in Fig. 4A, the three designs are all made of polydimethylsiloxane (PDMS) elastomer, but the first design has only a plain robot footpad (design 1) without any microstructures or coated bioadhesives, the second design (design 2) has only microstructures, while the third design (design 3) has both microstructures and are sequentially coated by hydrogel and bioadhesives. Figure 4B compares the friction of three different robot footpad designs on a porcine esophagus tissue surface ex vivo, characterized using a customized setup under a preload of 0.1 mN (figs. S6 and S7). Design 3 has the largest friction *f*_s_ (~ 2.2 mN) among the three pad designs. In contrast, design 1 shows a friction of about 0.5 mN, which leads to slipping. Notably, the inclusion of the microspikes increases the cross-sectional contact area and also yields the ploughing effect (*39*) in the process. The bioadhesives, on the other hand, also enhance the effective interfacial shear strength between the microspikes and the mucus layer by enlarging the bonding force via mucoadhesion. From the design perspective, we can adjust the friction between the robot footpad and the tissue surfaces by varying the microspike diameter *d*_s_ (Fig. 4C), the height ratio *h*_s_/*d*_s_, and the spacing ratio *l*_s_/*h*_s_ (fig. S9), where *h*_s_ and *l*_s_ represent the height and spacing of the spikes, respectively. We mainly design the size and geometric parameters of the spikes to control the contact area and penetration depth for both desired adhesion and friction. The designs are suboptimal because they could be further optimized to maximize the friction and range of adhesion, but they are sufficient to allow the soft robot to climb porcine gastrointestinal (GI) tract and respiratory tract tissues in our magnetic actuation system. Potentially, there exists an optimal design in terms of the spike distribution and size for desired friction and adhesion while minimizing the robot size. The optimal design depends on the targeted surface and requires efficient theoretical models or extensive experiments to complete the optimization, which we will explore in the future work.

In Fig. 4D, we compare the adhesion property of the three different robot footpad designs under different preloads on tissue surfaces from a porcine small intestine ex vivo. The green area represents a region where a desired adhesion-preload relationship is located. In this region, the footpad adhesion is sufficient to balance the gravity force of the robot (0.03 mN) and the carried payloads (up to 10 times the robot gravitational force). At the same time, the pad adhesion is still less than the maximum allowable preload so that the robot footpad could be peeled off from the substrate. Design 3 falls into the green region with a sufficient adhesion, ranging from 0.1 to 0.95 mN, with a preload varying from 0.1 to 1 mN. In contrast, design 1 yields an adhesion stronger than the preload, with a preload of 0.1 mN and 0.5 mN, making the robot footpad difficult to be peeled off. Design 2 shows a much weaker adhesion for about 0.2 mN even with a preload of 1 mN, making it difficult to climb vertically or invertedly, especially while carrying payloads. In addition, design 3 shows the largest adhesion range with Δ*f*_a_ = 1 mN, allowing a wide range of tunable adhesion compared with 0.5 mN for design 1 and 0.15 mN for design 2.

In addition, as a prominent advantage of using bioadhesives in design 3, the adhesion can also be controlled by varying the contact time (*26*). As shown in Fig. 4E, the adhesion of design 3 has significant dependence on contact time in contrast to the other two pad designs, allowing an adhesion range from 0.3 to 0.9 mN when the contact time varies from 1 to 10 s at a preload of 1 mN. This is because the bioadhesives could diffuse into the mucus layer and bond to the mucus via a cross-linking process, which are both time dependent (*25*). On the other hand, we could vary the contact time to further broaden the adhesion range by regulating the contact time of each robot footpad within a locomotion cycle, providing an extra control parameter to adapt to various tissue surfaces. In addition, we have conducted experiments to see the temporal degradation of the adhesion when repeatedly loading and peeling the robot footpads (see fig. S17). The degradation is less than 50% after *N* = 3000 cycles on all the GI tract tissues. This degradation may reduce the adhesion of the footpads, but we could compensate for it by increasing the preload force or contact time. In addition to the contact time and preload, the adhesion range of design 3 can also be intrinsically designed by varying the dimensional parameters of the microstructures such as diameters (Fig. 4F) and height ratios (fig. S9).

To further justify the effectiveness of the robot footpad design (design 3) for the climbing locomotion, we experimentally tested soft robots with the three different robot footpad designs as shown in Fig. 4G and movie S3. In the top row of Fig. 4G, with the same **B**(*t*), the robot footpads in design 1 tend to slip on the mucus-covered tissue surface rather than be peeled off. This is because the mucus layer is composed of water and mucins forming a lubricant layer between the robot footpads and the tissue surface. In contrast, the robot footpads in design 2 could avoid the slipping motion because the microspikes penetrate the entangled networks of the mucus and provide strong friction as shown in the middle row of Fig. 4G. However, these robot footpads have weak adhesion to the mucus layer, which makes the robot easy to fall off because of the reduced contact area between the robot footpads and the mucus layer. Last, when coated with a thin layer of hydrogel and chitosan sequentially on the microspikes, the robot footpads in design 3 have strong adhesion between the microspikes and the mucus, as well as sufficient friction to avoid slipping, as shown in the bottom row of Fig. 4G. With the robot footpads in design 3, the soft climbing robots could have inverted climbing on tissue surfaces from a porcine esophagus.

To show the advantage of the proposed robot footpad design (design 3) for controllable adhesion and friction on various tissue surfaces, we quantify the adhesion and friction of three robot footpad designs on the tissue surfaces from the GI tract and respiratory tract of a pig. In Fig. 4 (H and I), the robot footpad in design 3 shows the best performance among the three designs in terms of the maximum friction and the range of adhesion. This design combining both microspikes and bioadhesives can be generalized to other tissue surfaces. For example, the geometry of the microspike can be redesigned on the basis of the targeted surface properties. Figure S9 shows that by varying the geometric parameters of the microspikes, the adhesion between the pad and the tissue surfaces can be tuned to be 0.25 to 1.5 times the adhesion of the current design. Meanwhile, we could tune the density and the size of the microspikes, the concentration, and types of bioadhesives (*40*–*44*) for targeted tissue surfaces requiring different adhesion.

### Vertical and inverted climbing on versatile 3D tissue surfaces

**Fig. 5.**
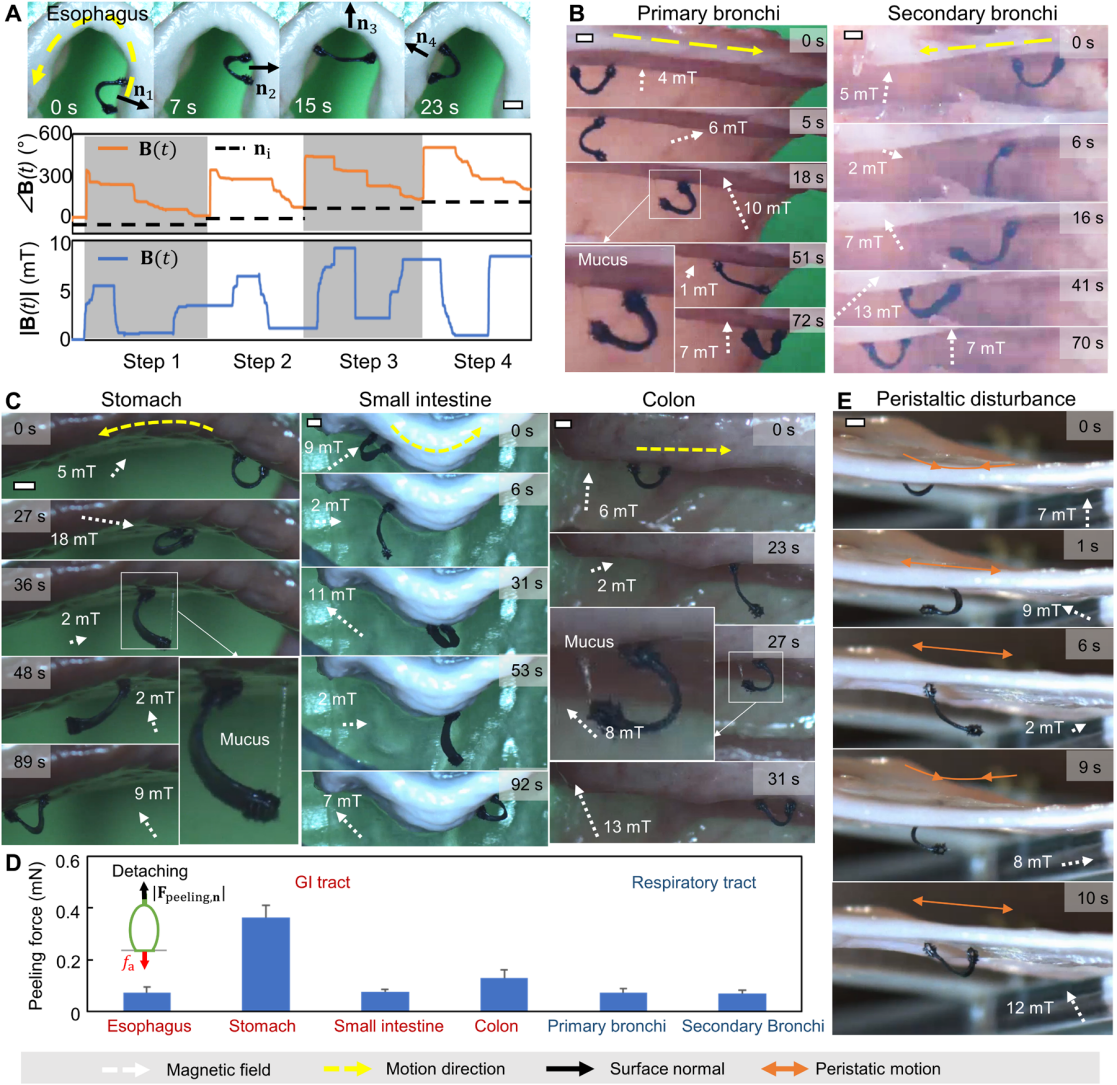
Vertical and inverted climbing versatile 3D porcine tissue surfaces ex vivo. (**A**) Video snapshots (top row; movie S4) and example magnetic fields (bottom row) of the robot climbing an inverse U–shaped porcine esophagus tissue surface. (**B**) Video snapshots (movie S4) of the inverted and axial climbing of the robot on tissue surfaces from a porcine respiratory tract (main and secondary bronchus of a porcine left lung). (**C**) Video snapshots (movie S4) of inverted climbing of the robot on dome-shaped, V-shaped, and tubular tissue surfaces from a porcine stomach, small intestine, and colon, respectively. The tissues were attached to a 3D printed phantom structure as a back layer. (**D**) Estimated peeling and loading forces on tissues due to the whole-body rotation. Estimation is based on the deflection curve of the deformed body, its original magnetization profile, and the applied magnetic field. Error bar represents the SD for *n* = 4 measurements. (**E**) Video snapshots (movie S4) of the robot withstanding peristatic disturbances while climbing a porcine small intestine tissue surface. In all figures, scale bars, 1 mm. Photo credit: Yingdan Wu and Xiaoguang Dong, Max Planck Institute for Intelligent Systems.

With the loading and peeling mechanisms, the soft-body deformation and the unique robot footpads, we show that our soft climbing robot could have vertical and inverted climbing on versatile 3D tissue surfaces from the porcine GI tract and respiratory tract with a wide range of reachability and maneuverability. For example, Fig. 5A illustrates how **B**(*t*) is adjusted to enable the robot to climb a curved porcine esophagus tissue surface. In this example, the robot climbs the curved surface with four selected waypoints with the corresponding surface normal angles β = −30°, 0°, 90°, and 150° in the *x-z* plane, respectively. By rotating **B**_0_(*t*) according to the direction of the surface normal, the resulting magnetic waveform **B**(*t*) can allow the robot to climb the highly curved tissue surfaces. In addition, to realize out-of-plane turning on 3D tissue surfaces, the robot could vary its climbing plane gradually by controlling the working plane of the applied **B**(*t*), where the out-of-plane component of **τ**_net_ would rotate the robot as shown in fig. S15.

Figure 5 (B and C) and movie S4 present that our robot has the capability of climbing various tissue surfaces including inclines, walls, and overhangs with complex geometries. First, the robot can have inverted and axial climbing on tubular tissue surfaces of various diameters, such as the porcine primary bronchi with a 13-mm diameter (Fig. 5B, left column) and the secondary porcine bronchi with a 4-mm diameter (Fig. 5B, right column). Second, Fig. 5C demonstrates that the robot climbs the stomach, small intestine, and colon tissues from the porcine GI tract. The switchable adhesion on all these tested tissue surfaces estimated by ∣*f*_a, n_∣ = ∣**τ**_net_∣/*d* is within the range of achievable adhesion for the robot footpads (Fig. 5D). Therefore, our robot has a great potential to traverse different 3D tissue surfaces in the GI tract and the respiratory tract. Last, Fig. 5E shows that our robot could also withstand disturbances from the peristaltic motions of the tissue surfaces (see fig. S4 for producing the peristaltic motion), which are common in the GI tract such as in the small intestine (*35*).

### Cargo delivery function while climbing complex terrains with multimodal locomotion

**Fig. 6.**
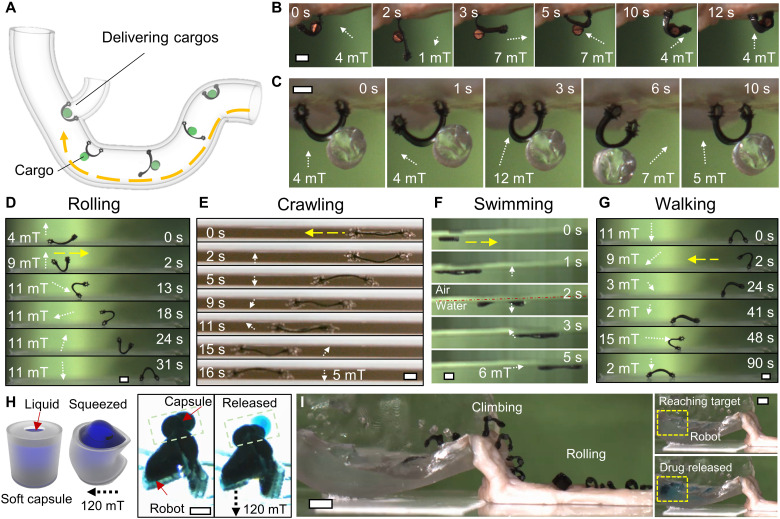
Cargo transport and delivery function of the robot while maintaining its soft-bodied multimodal locomotion capability. (**A**) Schematics of the soft climbing robot transporting payloads and tools while climbing complex 3D surfaces in enclosed and confined spaces. (**B**) Video snapshots (movie S5) of the soft climbing robot carrying a payload with high density and large body weight. The cargo is made of copper in a cylinder shape. It has a weight 20 times heavier than the robot weight. (**C**) Video snapshots (movie S5) of the soft climbing robot carrying a payload of high volume. The cargo is made of clear resin in a cylinder shape. It has a body volume three times the robot volume. (**D** to **G**). Video snapshots (movie S5) of the soft climbing robot achieving multimodal locomotion. (D) Rolling on solid substrates. (E) Crawling in narrow channels. (F) Swimming at the air-water interface. (G) Walking on solid substrates. (**H**) Schematics of a soft capsule and video snapshots (movie S5) of releasing liquid cargos by a soft capsule. (**I**) Overlapped video snapshots (movie S5) of the soft climbing robot navigating in complex 3D environments with multimodal locomotion, including rolling, climbing, and swimming, and completing liquid cargo delivery by carrying a soft capsule. Scale bars, 1 mm (B to H) and 5 mm (I). Photo credit: Yingdan Wu and Xiaoguang Dong, Max Planck Institute for Intelligent Systems.

The soft climbing robot could also carry cargos and navigate on complex 3D terrains that are difficult for other locomotion modes as shown in Fig. 6 and movie S5. As shown in Fig. 6A, it could climb complex 3D surfaces while carrying a payload. Figure 6 (B and C) shows that the robot could carry payloads up to 20 times its body weight and up to 3 times its body volume, respectively. Moreover, the soft climbing robots could still maintain the multimodal locomotion, such as rolling, climbing, and swimming, as shown in Fig. 6 (D to G). Figure 6D shows that the soft climbing robot could deform into a C or V shape and roll on the substrate surface by applying a rotating magnetic field. Then, Fig. 6E shows that the robot could also crawl between a narrow gap with a body traveling wave induced by a rotating magnetic field. The microspikes on the robot footpads allow the soft climbing robot to have a large friction to better crawl between slippery tissue surfaces (fig. S12). Meanwhile, the robot can walk by repeatedly changing its pivoting point and elongating or shortening its footpad distance as shown in Fig. 6F. Last, it could also swim at the air-water interface by creating a traveling wave upon applying a rotating magnetic field perpendicular to the air-water interface (Fig. 6G).

With the ability to carry payloads and maintain the multimodal locomotion, the soft climbing robot thus could carry various tools for advanced operations at locations that are impossible or very challenging for the medical tool itself to reach. For example, we demonstrate that the soft climbing robot carries a previously reported soft-bodied magnetic capsule (*45*) for liquid drug delivery. The schematics of the soft capsule are illustrated in Fig. 6H, where the soft capsule could deform upon applying a large magnetic field (~120 mT) and rapidly release the liquid “drug” emulated using water with a food dye. With the multimodal locomotion and especially the proposed climbing locomotion in this work, our soft climbing robot could negotiate a more complex 3D terrain while carrying a drug reservoir as shown in Fig. 6I. The robot could roll on a tissue surface for a fast motion and climb vertically to reach a location partially filled with water. After swimming across the air-water interface, it could eject the liquid cargo inside its carried drug reservoir for rapid liquid drug release on demand upon applying a large magnetic field. In the future, the soft climbing robot could also carry other medical tools such as miniature devices with spring-loaded microneedles (*46*) to penetrate deep tissues for delivering macromolecules, such as insulin, and thermally triggered microgrippers (*47*) to perform tissue biopsy.

### pH-triggered drug delivery function with potential long-term retention

**Fig. 7.**
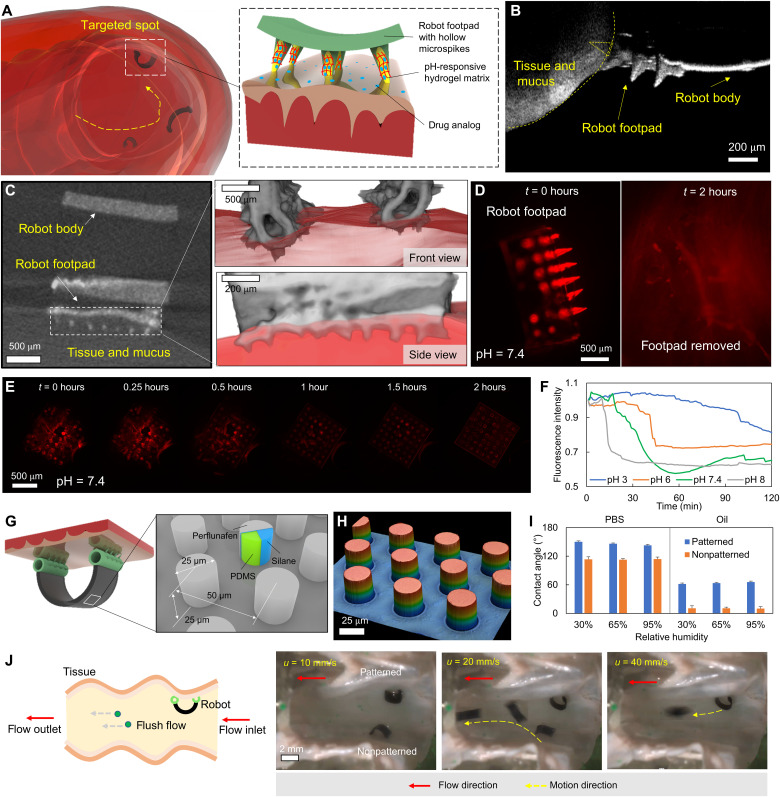
pH-triggered targeted drug delivery function by overcoming the mucus layer barrier with a potential long-term retention. (**A**) Schematics of reaching 3D disease spots in the GI tract and the on-demand drug delivery mechanism. (**B**) Optical coherence tomography (OCT) image of the robot footpad structures into the porcine small intestine mucus layer. (**C**) X-ray micro–computed tomography (microCT) image and 3D reconstruction of the robot footpad structure penetrating the porcine small intestine mucus layer. (**D**) Fluorescence images of the robot footpad structure penetrating into the porcine small intestine mucus layer and the mucus layer after removing the robot footpad structure after 2 hours. (**E**) Sequential snapshots during the swelling process of the robot footpad structure in a phosphate-buffered saline (PBS) buffer solution with a pH around 7.4. (**F**) The fluorescence intensity of the robot footpad structures in water solutions of different pH values as a function of time. (**G**) Illustration of the patterned robot body surface for reducing body adhesion and fluid drag. (**H**) The laser-optical scanning image of the patterned robot body surface. (**I**) The PBS and vegetable oil (rapeseed, K class; Kaufland AG) contact angles of the robot body surfaces with and without patterning under various relative humidity. (**J**) The schematics and video snapshots (movie S6) of soft climbing robots with and without the patterned body surfaces while staying on the tissue of a porcine small intestine ex vivo in a tubular structure with flushing waters of different speeds. Photo credit: Yingdan Wu and Xiaoguang Dong, Max Planck Institute for Intelligent Systems.

As another proof-of-concept medical function, in Fig. 7 and movie S6, we show that our robot could reach a target location in 3D that are difficult for other locomotion modes, penetrate deep into the mucus layers with a potential long-term retention, and deliver drug on demand by integrating pH-responsive hydrogels. The lumens of the human GI tract are covered with mucus layers in different thicknesses and pH values varying from 2.0 to 7.4 (*48*). The mucus, a viscoelastic gel, consisting of water and mucin, which is the main dry weight component, is difficult to penetrate, preventing oral drug delivery to the surface of the capillaries when a disease such as ulcer develops (*49*). The ability to reach challenging disease spots and deliver drugs by overcoming the mucus barrier could allow more precise and effective treatment of diseases in lumens with mucus-covered tissues such as the small intestine of the GI tract. Here, we integrate a pH-responsive hydrogel to carry and release drugs and test their performance particularly on a porcine small intestine tissue ex vivo as it is similar to the human small intestine (*35*).

To allow the drug delivery function, we design hollow microspikes to replace the solid microspikes on the robot footpads. The hollow microspikes can contain hydrogel loaded by drugs inside the hollow space while maintaining the spike geometry for the penetration purpose. As shown in Fig. 7A, the hollow microspikes could be loaded with pH-responsive hydrogels and still promise the desired adhesion and friction properties (movie S6 and fig. S10). As we target drug release in the small intestine, we select a hydrogel called *P(NIPAm-co-AA)*, which could swell at a pH around 7.4 (*50*). The basic drug release mechanism is that when the hollow spikes penetrate the mucus layer with a pH larger than 7, the NIPAm-AA swells and releases the drug from the hollow spikes to the tissue surfaces and the surrounding mucus layers. In addition, we also show that even the swollen microspikes on the footpads could allow the climbing motion of the robot on tissue surfaces at a temperature and humidity level similar to that of the human body (see movie S7 and fig. S20).

First, to verify the performance of penetrating mucus layers, in Fig. 7 (B and C), we use an optical coherence tomography (OCT) machine and a micro–computed tomography (microCT) imaging system to visualize and measure the penetration depth (~50 μm) into the mucus layer of a porcine small intestine tissue by the hollow spikes on the robot footpads. As the porcine small intestine mucus layer has an average thickness of about 50 μm (*24*), the penetration is sufficiently deep to reach the tissue surface. For other disease spots with a thicker mucus layer, the penetration depth could be further increased by a stronger preload to allow a penetration depth up to 200 μm.

Second, to quantify the drug release performance, we use fluorescence particles to simulate the drug molecules and perform controlled experiments to compare the drug release speeds at different pH values. We load fluorescence particles into the *P(NIPAm-co-AA)* matrix by mixing them with the monomer solution and cross-linking the structure in the hollow spikes. The fluorescence particles could be visualized under a fluorescence microscope to observe and quantify the drug release performance. Figure 7D shows the fluorescence microscope image of the drug-loaded hollow spikes on the robot footpad penetrating into the mucus layer of a porcine small intestine tissue and the fluorescence microscope image of the mucus layer after 2 hours with the robot footpads removed. The distribution of the fluorescence particles clearly shows the effectiveness of the pH-triggered drug release mechanism. We also show that the swelling speed is strongly pH dependent in Fig. 7 (E and F) and fig. S11, indicating the drug release will only be triggered substantially in mucus layers with a pH value larger than 7.

Last, the design with both the strong-adhesive footpad and the weak-adhesive body (Fig. 7G) allows the robot to stay at a target location for a relatively long time (hours) because it can resist external disturbances such as vibration and bodily fluids. We pattern the robot body with an omniphobic structure (*51*) to allow weak adhesion to both water-based and oil-based liquids. As shown in Fig. 7H, the robot body has micropillar structures with coated *(heptadecafluoro-1,1,2,2-tetrahydrodecyl)trichlorosilane* (Sigma-Aldrich Inc.) and *perfluorodecalin* (Sigma-Aldrich Inc.) based on the protocol in (*51*) such that it has water and oil contact angles notably larger than that of a plain robot body without any coating (Fig. 7I). Figure 7J shows that the robot with an omniphobic body could resist a flushing water flow up to 40 mm/s larger than the maximum GI tract fluid flow at 20 mm/s (*52*), in contrast to only 10 mm/s for a robot without such a body surface design. Our robot thus could be potentially controlled to locomote on the GI tract tissue surfaces guided by medical imaging, penetrate deep into the mucus layers with a potential long-term retention, and deliver drugs on demand. As future work, we will study the maximum duration of adhering to the tissue surfaces by our robot while withstanding fluid flows and other disturbances.

## DISCUSSION

In summary, we have developed an untethered millimeter-scale soft climbing robot that is capable of climbing complex 3D surfaces. We have developed a generic peeling-and-loading mechanism for controllable adhesion using both the soft-body deformation and whole-body motion of the robot. This generic mechanism is implemented using different adhesive robot footpad designs, allowing the vertical and inverted surface climbing of the soft robot on diverse 3D surfaces with complex geometries, different surface properties, and different softness. We have further demonstrated such a mechanism to create a soft robot for climbing soft tissues. By integrating both microstructures and tough bioadhesives into a unique robot footpad design, we have demonstrated controlling the soft climbing robot to locomote freely on 3D tissue surfaces from the porcine GI tract and respiratory tract. As potential functions, the robot has been shown to deliver cargos while climbing in enclosed small spaces and still maintain multimodal locomotion, including walking, rolling, and swimming, allowing a broader reachable workspace in complex 3D terrains. With the ability of climbing complex 3D tissue surfaces, we have demonstrated a potential medical function of the climbing robot as a tool to overcome the mucus barrier and release the drug in a porcine small intestine ex vivo by integrating pH-triggered hydrogels into the microspikes.

The proposed peeling-and-loading mechanism is generic and could work with other microstructure designs and bioadhesives. For example, with the same loading and peeling mechanisms, our robot could potentially climb tissues covered by the mucus of different thicknesses by designing the height ratio and other dimensional parameters of the microstructures. The robot could also potentially climb various wet tissue surfaces such as heart surface tissues not covered by a mucus layer (fig. S13) by further integrating diverse wet bioadhesives (*40*–*44*) into the robot footpads. So far, it is still challenging for our robot to climb tissue surfaces under water. For example, fig. S14 shows that our robot climbs a porcine small intestine tissue under water with ultrasound imaging guidance. However, the robot cannot climb for a long distance, as the adhesion between the robot footpad and the tissue surface is weak and will diminish quickly. In the future, with more advanced underwater bioadhesives (*53*) to be developed, our robot could potentially climb 3D tissue surfaces fully submerged under water. Nonetheless, in the application scenario where the soft climbing robot is fully submerged under liquids, a swimming locomotion (*7*, *9*, *10*) could be used for navigating through the fluidic environment. The ability of climbing 3D tissue surfaces while still maintaining the multimodal locomotion enriches the negotiable terrains for wireless miniature soft robots.

Toward in vivo clinical applications, our soft climbing robots have to be further localized using medical imaging modalities, such as ultrasound imaging (fig. S14) and x-ray fluoroscopy (*12*). The constraints of imaging resolution, speed, contrast of the robot materials and the compatibility with the actuation method need to be simultaneously considered (*54*) for choosing the appropriate medical imaging methods. To allow the robot to access target locations in the shortest durations, the robot could be delivered by capsule endoscopes (*55*) or continuum robots (*54*) first and then locomote by itself to the locations not achievable or very risky for other medical tools. In the respiratory tract, it is also important to retrieve the robot with the help of other medical tools. For the GI tract, the robot could get out of the human body through the digestive passage after completing its tasks.

To scale up or down the robot size for various applications, we have to consider the scaling laws in the robot physics (*56*). We consider three key components of the robot physics, namely, gravity, adhesion, friction, and net magnetic torque. On the basis of a scaling analysis (see the “Scaling analysis of the climbing robot” section of the Materials and Methods), the scaling law of the gravity force *G*, adhesion *f_a_*, friction *f_s_*, and net magnetic torque τ_net_, in terms of the robot body length *L*, are given by *G* ∝ *L*^3^, *f*_a_ ∝ *L*^2^, *f*_s_ ∝ *L*^2^, and τ_net_ ∝ *L*^3^*M*∣*B*∣. Meanwhile, the scaling law of the loading and peeling forces are given by *F*_peeling_ (or *F*_loading_) ∝τ_net_/*d* ∝ *L*^2^. These scaling analyses suggest that it would be preferred to scale down the robot size with the current design for climbing rather than scaling up because of the gravity effect. The peeling-and-loading mechanism is generic regardless of the robot size as the peeling and loading forces are comparably scaling with the robot size similar to the adhesion and friction. However, without any compensation of the gravity, it would be preferred to scale down rather than scale up the robot size to minimize the gravity effect. Otherwise, active compensation for the robot weight needs to be considered using stronger adhesives and powerful actuators to generate stronger peeling and loading forces. Nonetheless, our work provides a proof-of-concept demonstration, which could potentially inspire untethered soft climbing robots at various length scales, although a further optimization is required for achieving desired peeling and loading behaviors.

Despite the demonstration of the soft climbing robot using magnetic actuation, the same peeling-and-loading mechanism, the robot kinematics, and the robot footpad designs could be broadly applied to or inspire a variety of soft robot designs using different actuation methods. For example, untethered soft climbing robots could be actuated by various off-board actuation methods, such as using liquid crystal elastomer actuators driven by temperature (*57*), light, chemical, and other stimuli (*58*), as well as on-board actuation methods, such as using dielectric elastomer actuators (*59*) and electrically controlled shape memory actuators (*60*) driven by electric voltages. The generic peeling and loading mechanisms could be reproduced if a whole-body torque could be applied on the robot such that the peeling and loading forces could be induced when the integrated adhesive robot footpads sequentially adhere to the 3D surfaces. At the same time, the robot symmetry-breaking motions could be realized by programmable soft-body deformation by encoding specific material properties such as anisotropic stiffness (*57*) and controlling the actuation signal sequences (*59*). Our proposed method thus would also inspire a wide range of untethered miniature soft robots capable of climbing complex and unstructured terrains in 3D confined and enclosed spaces for potential applications in environmental inspection, space exploration, and other applications.

## MATERIALS AND METHODS

### Fabrication of the soft climbing robot

The soft climbing robot is composed of a ferromagnetic-elastic sheet with patterned surface properties and two ring-shaped elastic footpads with bioadhesive-coated microspikes. The fabrication process consists of the following steps.

### Fabrication of the ferromagnetic-elastic robot body

First, Ecoflex 00-30 silicone rubber (Smooth-On Inc.) and NdFeB microparticles (average diameter, 5 μm; MQFP-15-7, Neo Magnequench) were mixed at a 1:2 ratio by weight and then poured onto a poly(methyl methacrylate) substrate with 150-μm-thick spacers, against which a razor blade was scraped for the control of the sheet thickness (fig. S1A). The scraped mixture was cured at 90°C on a hot plate for 30 min. The cured sheet was then cut into a 4 mm–by–2 mm rectangular sheet using a laser machine (LPKF ProtoLaser U3, LPKF Laser & Electronics AG) as shown in fig. S1B. The material has a density of 2.5 g/cm^3^ and a Young’s modulus of 163±5 kPa measured by a tensile testing machine (5940 series, Instron GmbH).

Then, the omniphobic surface patterning and coating (movie S6 and Fig. 7) were conducted as explained in the following steps. An SU-8–positive mold on a silicon wafer was prepared using photolithography (Mask aligner MJB4, SÜSS MicroTec Lithography GmbH), which has a hexagonally distributed matrix of holes of 25 μm in depth, 25 μm in diameter, and 50 μm in spacing distance. PDMS solution (SYLGARD 184, Dow Inc.) with a weight ratio of 10:1 between the monomer to the cross-linker (denoted as 10:1 PDMS in the following text) was poured onto the positive mold and then degassed for 1 hour. The top surface of the mold was then scraped by a razor blade for removing the redundant PDMS solution. The ferromagnetic-elastic rectangular sheet prepared in the previous step was then placed on top of the mold with one side facing down and was pressed with gentle pressure (fig. S1C). The PDMS solution was cured at 90°C on a hot plate for 1 hour. The sheet was peeled off from the mold with one side of the sheet patterned with micropillars. We repeated the above procedures to pattern the other side of the sheet with micropillars. Both sides of the sheet were then coated with *(heptadecafluoro-1,1,2,2-tetrahydrodecyl)trichlorosilane* (Sigma-Aldrich Inc.) and *perfluorodecalin* (Sigma-Aldrich Inc.) following the same protocol as in (*51*). The obtained omniphobic sheet was then cropped into a 3.7 mm–by–1.5 mm rectangular sheet using the laser machine. The two patterned surfaces of the sheet were subsequently rusted at the edges to remove the coated materials for easy bonding to the robot footpads. Last, the cropped rectangular sheet was wrapped around a 3D printed rod of 1.2 mm in diameter and 5 mm in length and magnetized in a uniform magnetizing field of 1.8 T (EZ7 VSM, MicroSense LLC) with a specific orientation where its two ends face the north pole (fig. S1D). The magnetized robot body has a magnetization of 61.9 kA/m.

### Fabrication of the robot footpads

The plain ring-shaped footpad (design 1 as shown in movies S1 and S3 and Figs. 3 and 4) was made from a 1.5 mm–by–1.5 mm–by–60 μm PDMS (monomer versus cross-linker mixing ratio: 20:1, denoted as 20:1 PDMS in the following text) patch, while the footpads with microstructures were prepared by molding. The mushroom-shaped gecko-inspired dry adhesive mold has hexagonally distributed mushroom fibers of 20 μm in stem diameter, 15 μm in stem height, 30 μm in plate diameter, and 5 μm in plate height and a 1.5 mm–by–1.5 mm–by–60 μm backing layer (design 2 for dry surfaces as shown in movie S2 and Fig. 3). The spike mold has a 7 by 7 conical spike matrix of 200 μm in height, 100 μm in diameter, and 200 μm in spacing and a 1.5 mm–by–1.5 mm–by–60 μm backing layer (design 2 for tissue surfaces as shown in movie S3 and Fig. 4). The hollow spike mold is based on the aforementioned spike mold with 20-μm eccentric through-holes with diameter of 40 μm on each spike (footpad for drug delivery as shown in movie S6 and Fig. 7). All these molds mentioned above were prepared using a two-photon polymerization (2PP) 3D printer (Photonic Professional GT, Nanoscribe GmbH) with a rigid commercial photo resin (IP-S, Nanoscribe GmbH) as shown in fig. S2A. After 2PP 3D printing, the master molds were developed in propylene glycol monomethyl ether acetate (Sigma-Aldrich Inc.) for 40 min and rinsed in isopropyl alcohol for 3 min. Subsequently, the molds were exposed to plasma (Tergeo Plasma Cleaner, PIE Scientific LLC) for 2 min for surface activation and then placed in a vacuum desiccator for 40 min with 0.2 ml of *(heptadecafluoro-1,1,2,2-tetrahydrodecyl)trichlorosilane* in a glass vial. The molds were then baked at 90°C in an oven for 40 min. The 10:1 PDMS solution was poured onto the mold and then degassed for 1 hour. After curing at 90°C in an oven for 1 hour, the negative molds were peeled off from the two master molds (fig. S2B). We repeated the above plasma, salinization, and baking processes for the two negative molds. The 20:1 PDMS solution was poured onto the negative substrate molds and then degassed for 1 hour. Before curing at 90°C in an oven for 1 hour, the redundant PDMS solution was removed by scratching the top surface of the negative molds using a razor blade. The cured mushroom fibers, the solid spike patch, and hollow spike patch were peeled off from the molds separately (fig. S2, C and D).

### Coating the robot footpads

To coat the hydrogel layers on top of the microspikes, benzophenone solution [20 weight % (wt%) in water; Sigma-Aldrich Inc.] was pipetted over the patches to initiate the surface bonding to hydrogels by serving as the hydrophobic photoinitiator. We implemented different hydrogels onto the two footpads for different functions including poly(ethylene glycol) diacrylate (Sigma-Aldrich Inc.) in the solid spike patch as a dissipative layer for bioadhesives and the pH-sensitive hydrogel P(NIPAm-co-AA) for the hollow spike patch as a matrix to carry microparticles. For the solid spikes, poly(ethylene glycol) diacrylate was poured onto the spike patch and then degassed for 30 min. After curing in a 365-nm ultraviolet (UV) chamber (ELG100S, Dinies Technologies GmbH) for 6 min, the coated patch was washed using deionized water to remove the unreacted chemicals (design 3 for tissue surfaces as shown in movies S3 to S5 and Figs. 4 to 6). For the hollow spikes, the P(NIPAm-co-AA) solution was prepared using 0.1 g of NIPAm (97%; Sigma-Aldrich Inc.) and 5 g of AA (99%; Sigma-Aldrich Inc.) as a monomer, 0.05 g of *N*,*N*′-methylenebis(acrylamide) (99%; Sigma-Aldrich Inc.) as a cross-linker, and 0.025 g of 2,2-dimethoxy-2-phenylacetophenone (99%; Sigma-Aldrich Inc.) as a photoinitiator. After the mixture solution was homogeneous by stirring, fluorescent particles (CellTracker Deep Red Dye; molecular mass of 698.3 Da; Invitrogen), as emulated drugs, were added at 1 wt% in the mixture solution. The hollow spike patch was then placed into the negative PDMS spike mold. Then, the fluorescent particles and P(NIPAm-co-AA) mixture was poured onto the mold and degassed for 30 min until the holes were filled with the solution (fig. S2E). Afterward, the negative spike mold with the hollow spike patch was sandwiched by two glass slides with four clippers to remove the redundant hydrogel solution and then cured in a 365-nm UV chamber for 6 min (fig. S2F). Deionized water was pipetted over the top surface of the mold for removing the unreacted chemicals and also for an easy release of the patch from the mold (footpad for drug delivery as shown in movie S6 and Fig. 7). To integrate the bioadhesive onto the pads, a bridging polymer chitosan (high molecular weight; Sigma-Aldrich Inc.) was dissolved into the compound MES buffer (Sigma-Aldrich Inc.) at a weight ratio of 2.0%, and the pH of the solution was adjusted to about 6. The unsulfated *N*-hydroxysuccinimide (98%; Sigma-Aldrich Inc.) was used as a coupling reagent with a concentration of 12 mg/ml in the final solution. Last, the bioadhesive solution (~100 μl) was applied to the surface of the pad (1.5 mm by 1.5 mm). The fabricated robot footpads were bonded to the two sides of the robot body using Ecoflex 00-30 as shown in fig. S2H.

### Magnetic actuation setup

As shown in fig. S3, the customized setup consists of three pairs of solenoids for generating uniform magnetic fields up to 30 mT within a 2 cm–by–2 cm–by–2 cm workspace. The workspace of our current magnetic actuation system for proof of concept is relatively small, but our climbing robot could be actuated by various existing magnetic actuation systems (*61*, *62*) with a relatively large workspace. In the future, we would develop an electromagnetic actuation system with a larger workspace to cover a large portion of the animal and human bodies. The setup was controlled using NI compactRIO embedded controller (National Instruments) connected to a PC. A driver board with eight motor drivers (SyRen 25, Dimension Engineering) was used to actuate the solenoids powered by a power supply. The cameras (Blackfly S USB3, FLIR Systems) were connected to the same PC for visualizing and recording the experimental videos simultaneously. The module to control the relative humidity and temperature of the workspace (fig. S19, A and B) was used for the climbing test under controlled relative humidity and temperature as shown in movie S7. The samples were placed in a 1.92-liter L-shaped acrylic box where the temperature and relative humidity were controlled by a heater (LM-Standard, RO/SE Blechverarbeitung GmbH & Co. KG) and an air humidifier (Emma, Stadler Form GmbH), respectively. A hygrometer (Voltcraft HY-10, Voltcraft Engineers Private Limited) was used to monitor the relative humidity and the temperature inside the box.

### Characterization of the friction and adhesion of robot footpads

The friction and adhesion tests were conducted on a customized experimental setup as shown in fig. S6. The adhesion and friction were measured using two load cells (GSO-25, Transducer Techniques LLC) connected to the probe vertically and horizontally, respectively. The load cells were mounted on a linear motor stage that can translate vertically. The specimen was placed on a glass slide mounted on a linear motor as a tangential translation stage. A camera with a microscope was placed below the glass stage for visualizing the contact process. The process of the friction test was composed of loading, traveling, and retracting steps, where the traveling step was achieved via the tangential translation of the specimen stage. The process of adhesion test consisted of the loading and retracting steps, both of which were only involved with the vertical motion of the probe. The robot footpads tested in the experiments included the two robot footpad designs for dry surfaces as shown in Fig. 3, the three robot footpad designs for tissue surfaces as shown in Fig. 4, the unique robot footpads with coated bioadhesives and various geometries as shown in fig. S9, and the robot footpads loaded with drug analog as shown in fig. S10. They were attached to a probe and aligned to the substrate as shown in fig. S7. The tissue samples were prepared for every 10 tests (within 5 min) from the fresh porcine esophagus, stomach, small intestine, colon, primary bronchi, and secondary bronchi to avoid the dehydration of the tissues. For the friction test, the approaching and retracting speeds of the probe were set to 50 μm/s, while the preload and contact time were 0.1 mN and 5 s, respectively. The tangential displacement and speed were 0.5 mm and 50 μm/s, respectively. For the adhesion test, the approaching and retracting speeds of the probe were set to 50 μm/s, while the preload and contact time were varied as reported in Fig. 3E and fig. S8. The measurement of adhesion under loading-peeling cycles in fig. S17 was conducted using the additional module for controlling the relative humidity and the temperature as shown in fig. S19D.

### Characterization of the penetration depth of microspikes on robot footpads

The penetration depth of the microspikes on the small intestine tissues was characterized using the OCT (ThorImageOCT; Thorlabs Inc.) and the x-ray microCT (Bruker Skyscan 1276; Bruker Inc.) systems as reported in Fig. 7 (B and C). The robots were loaded onto the small intestine tissue with a magnetic field of about 20 mT. To improve the contrast of the microspikes in the mucus layer during the microCT imaging, the density of the footpad microspikes and the body of the robot was increased using a mixture of Ecoflex 00-30 and NdFeB particles with weight ratios of 6:1 and 4:1, respectively.

### Characterization of the drug delivery function of the hydrogel microspikes

The fluorescent microparticles (CellTracker Deep Red Dye, with a molecular mass of 698.3 Da; Invitrogen) were mixed with NIPAm-co-AA and cured on the robot footpad. For the characterization of releasing the fluorescent microparticles, the footpads were fully submerged in various standard phosphate-buffered saline (PBS) solutions with a pH value of 3, 6, 7.4, and 8 (fig. S11) or loaded on small intestine tissues. The samples were then placed under a fluorescence microscope (Nikon Inverted Microscope Eclipse Ti-E, Nikon Inc.), and the image fluorescence intensities (band-pass emission, 670 nm) were analyzed using the Nikon software. The obtained images were postprocessed using customized codes in MATLAB 2020a (MathWorks Inc.) for the plots of the fluorescence intensities as a function of time in Fig. 7F.

### Characterization of the surface properties of the robot body

The contact angle measurement in Fig. 7I was conducted in a commercial contact angle measurement machine (Drop Shape Analyzer DSA, Krüss GmbH) using the sessile drop mode. The liquids used include standard PBS solution and vegetable oil (rapeseed, K class; Kaufland AG). The polymer samples tested included a 10 mm–by–5 mm ferromagnetic-elastic sheet without patterned surfaces and a 10 mm–by–5 mm patterned ferromagnetic-elastic sheet. The tissue samples, including fresh tissues from porcine esophagus, stomach, small intestine, colon, primary bronchi, and secondary bronchi, were cut into 5 mm–by–5 mm patches and spread to be smooth. For each trial in the test, the contact angle was measured at least five times with a fixed droplet volume of 5 μl. All measurements were performed at the temperature of 37°C and relative humidity of 30, 65, and 95%, achieved via the additional module for controlling the relative humidity and the temperature as shown in fig. S19C. The ability of the climbing robots to withstand a flushing flow was further tested in a customized setup as shown in fig. S5. The small intestine tissues attached to the corresponding phantom model were placed in a glass tube with an inner diameter of 25 mm, while its two ends were sealed and connected to two rubber tubes with an inner diameter of 7 mm. One rubber tube as an inlet was connected to a 100-ml syringe mounted on a syringe pump, while the other one, as an outlet, was connected to a water tank to collect the water. We used plastic green spherical particles (200 μm in diameter) in the water to help track the flow speed. The robots with the plain body and the patterned body were loaded onto the small intestine tissue in the middle of the glass tube at similar locations so that both robots experienced the same flushing fluid flow. The speed of the flushing fluid flow was adjusted via the syringe pump.

### Preparation of porcine organs and phantom models

The fresh porcine esophagus, stomach, small intestine, colon, and left lungs were purchased from the local food factory, Ulm, Germany. The organs were emptied and minimally cleaned by water without damaging the mucus layer. The human GI tract phantom models were purchased from a medical phantom company (Trandomed 3D Medical Technology Co.) and cropped in SolidWorks 2020 (Dassault Group). The 3D phantom models were prepared using a 3D printer (Form 3, Formlabs Inc.) with the photo resin Elastic 50A (Formlabs Inc.). Porcine tissues were cut and attached to human phantoms in the experiments. All tests and characterizations were conducted on fresh tissues within 24 hours (stored in a fridge at 2°C).

### Scaling analysis of the climbing robot

We compared the scaling effect of the peeling and loading forces, gravity, adhesion, and friction applied on the robot body and footpads (fig. S21). First, we define *PA* to quantify the relative scaling of the peeling (or loading) force *F*_peeling_ (*F*_loading_) and the total adhesion *f*_a_ applied on the whole robot, which is given by

$$\begin{array}{c} \mathrm{PA}=\frac{F_{\text{peeling}}}{f_{a}}=\frac{|\tau_{\text{net}}|/d}{\sigma_{a}A_{c}}=\frac{|\frac{1}{d}[\int_{0}^{L}M(s)\;\mathrm{wt}ds]\times B|}{\sigma_{a}A_{c}}\\ \propto \frac{L^{-1}\cdot {\mathrm{ML}}^{3}B}{\sigma_{a}L^{2}}\propto \frac{\mathrm{MB}}{\sigma_{a}} \end{array}$$ (1)

where the footpad distance *d* is proportional to *L* and the contact area *A*_c_ scales as *L*^2^. Equation 1 shows that the peeling and loading forces scale similarly to the adhesion as the robot length *L* scales up or down. It suggests that our peeling-and-loading mechanism using net magnetic torques could still overcome the interfacial adhesion when the robot size is smaller or larger.

Second, we define *GA* to quantify the relative scaling of the gravity force *G* and the total adhesion *f*_a_ applied on the whole robot, which is given by

$$\mathrm{GA}=\frac{G}{f_{a}}=\frac{(\rho_{b}\mathrm{Lwt}+\rho_{f}w^{2}t_{f})g}{\sigma_{a}A_{c}}\propto \frac{(\rho_{b}L^{3}+\rho_{f}L^{3})g}{\sigma_{a}L^{2}}\propto L$$ (2)

where ρ_b_, ρ_f_, *t*_f_, and σ_a_ denote the robot body density, the footpad density, the footpad thickness, and the interfacial adhesion strength, respectively. Equation 2 indicates that the interfacial adhesion strength needs to be increased to overcome the robot weight as *L* increases or vice versa.

Last, we define *GF* to quantify the relative scaling of the gravity force *G* and the total friction *f_s_* applied on the whole robot, such as in the vertical climbing case. *GF* is given by

$$\mathrm{GF}=\frac{G}{f_{s}}=\frac{(\rho_{b}\mathrm{Lwt}+\rho_{f}w^{2}t_{f})g}{\tau_{a}A_{p}}\propto \frac{(\rho_{b}L^{3}+\rho_{f}L^{3})g}{\tau_{a}L^{2}}\propto L$$ (3)

where *A*_p_ and τ_a_ denote the cross-sectional area of the part of the robot footpad in contact with the substrate and the interfacial shear strength, respectively. Equation 3 indicates that the friction needs to be increased to overcome the robot weight as the robot length *L* increases or vice versa.

## Supplementary Materials

### This PDF file includes:

Figs. S1 to S21 Supplementary Note References
