# Supplementary material for: Wireless soft millirobots for climbing three-dimensional surfaces in confined spaces
Source: Sci Adv. 2022 May 27;8(21):eabn3431. doi: 10.1126/sciadv.abn3431 (PMC9140972; doi:10.1126/sciadv.abn3431)
Supplement: Supplementary file 1 — Figs. S1 to S21 Supplementary Note References [file sciadv.abn3431_sm.pdf]

Science Advances
AAAS

Supplementary Materials for

Wireless soft millirobots for climbing three-dimensional surfaces in confined spaces

Yingdan Wu *et al.*

Corresponding author: Metin Sitti, sitti@is.mpg.de

Sci. Adv. **8**, eabn3431 (2022)
DOI: 10.1126/sciadv.abn3431

The PDF file includes:

Figs. S1 to S21
Supplementary Note
Legends for movies S1 to S7
References

Other Supplementary Material for this manuscript includes the following:

Movies S1 to S7

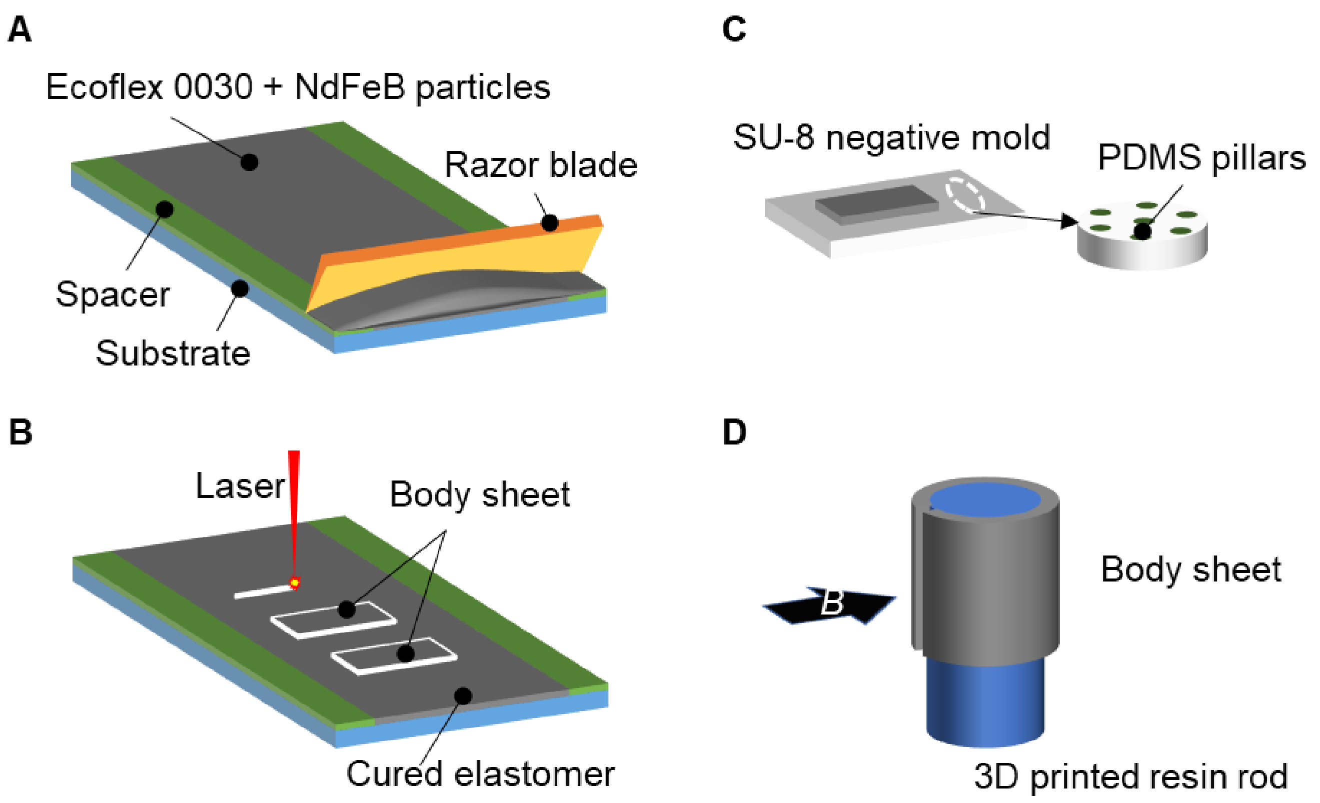

Fig. S1. Schematics of the fabrication process of the robot body. A. Illustration of fabricating a ferromagnetic-elastic sheet with a desired thickness. The mixture of Ecoflex-0030 elastomer and NdFeB microparticles were poured onto a PMMA substrate. A razor blade was used to scratch against the spacer for a uniform thickness. **B.** Illustration of the laser machining process to produce the robot body. The ferromagnetic-elastic sheet was cut into rectangular sheet with specified dimensions using a laser machine (LPKF ProtoLaser U3, LPKF Laser & Electronics AG). **C.** Illustration of the transferring molding process to fabricate PDMS micro-pillars for omni-phobic surface patterning. The micro-pillars on the body surface were fabricated using molding techniques. **D.** Schematic of the magnetization process for the ferromagnetic-elastic sheet of the robot body. The trimmed rectangular sheet was wrapped over a 3D printed cylindrical rod of 1.2 mm diameter and 5 mm length and then placed into a uniform magnetizing filed of 1.8 T (EZ7 VSM, MicroSense LLC) with the two ends facing to the north pole.

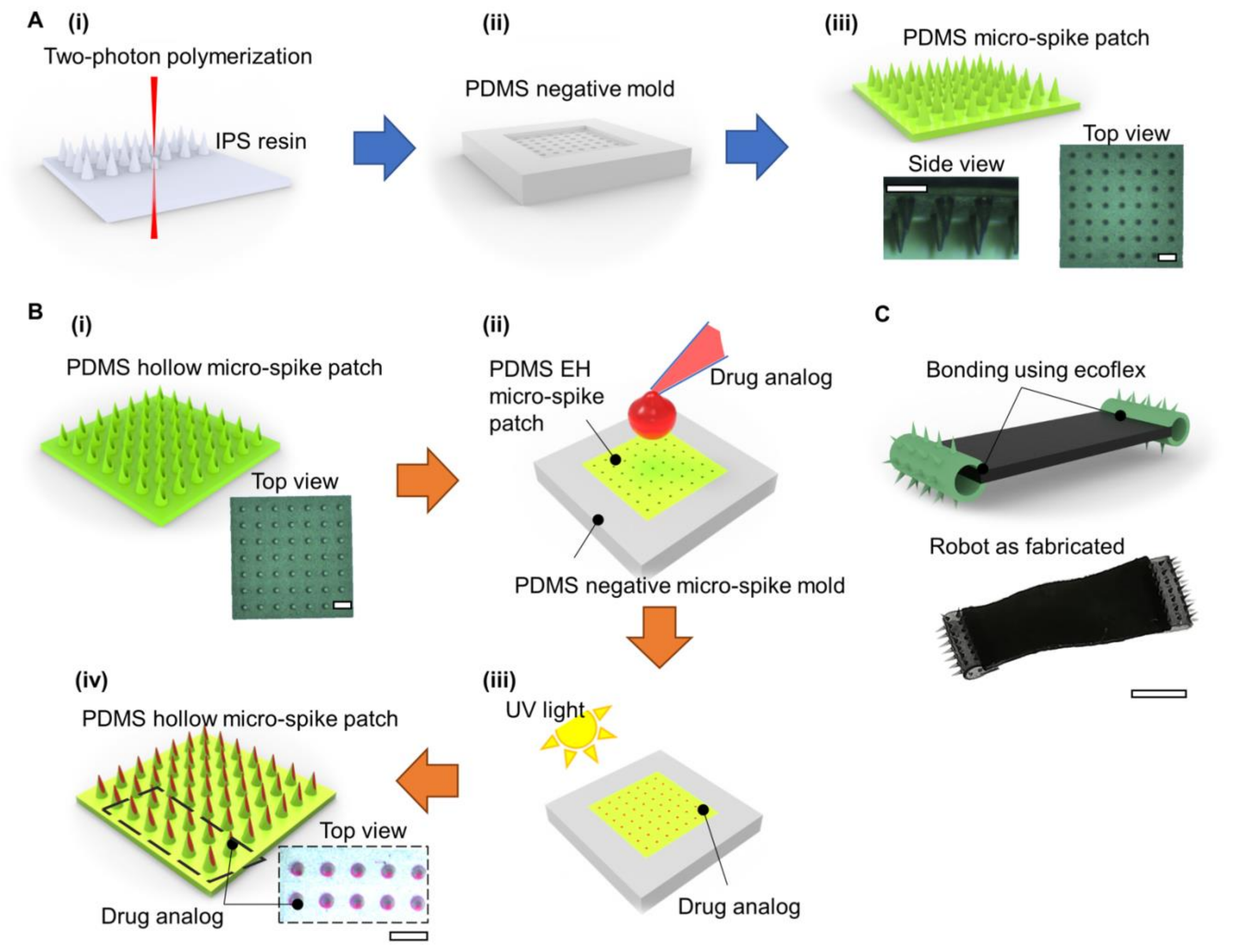

Fig. S2. Schematics of the fabrication process of the robot footpads. A. Illustration of the fabrication of the robot pad. **(i)**. Schematic of 3D printing the master mold using the Two-Photon-Polymerization (2PP). The master molds of the mushroom-shaped gecko-inspired dry adhesive, micro-spikes, and hollow micro-spikes were prepared using a 2PP 3D micro-printer (Photonic Professional GT, Nanoscribe GmbH) with a rigid IP-S commercial photoresist (Nanoscribe GmbH). **(ii).** Illustration of the PDMS negative mold for both the dry adhesive pad, the micro-spike pad, and the hollow micro-spike pad. These molds were obtained by molding 10:1 PDMS solution against the post-processed 3D printed IP-S master molds. **(iii).** Illustration of the 20:1 PDMS micro-spike pad patch. This patch was fabricated by molding 20:1 PDMS solution against the post-processed PDMS negative molds. **B.** Illustration of the fabrication of the hollow micro-spike pad with drug analog loaded. **(i).** The schematic of the 20:1 PDMS hollow micro-spike pad patch. **(ii)**. Illustration of the process of loading the PNIPAm-co-AA and fluorescence particles into the holes of a hollow micro-spike patch. After the hollow micro-spike patch was fitted into the PDMS negative micro-spike mold, the solution of the PNIPAm-co-AA and fluorescence particles was pipetted over the mold. **(iii).** Illustration of the process of curing the mixture of PNIPAm-co-AA and fluorescence particles in a 365 nm UV chamber. **(iv).** Illustration of the hollow micro-spike pad patch loaded with fluorescence particles. **C.** Illustration of the assembly process of the robot pads and the robot body and the corresponding soft climbing robot as fabricated. The body and pads were bonded using Ecoflex-0030 after the robot pads are coated by bioadhesives. Scale bars: **A** and **B**, 0.2 mm; **C**, 1 mm.

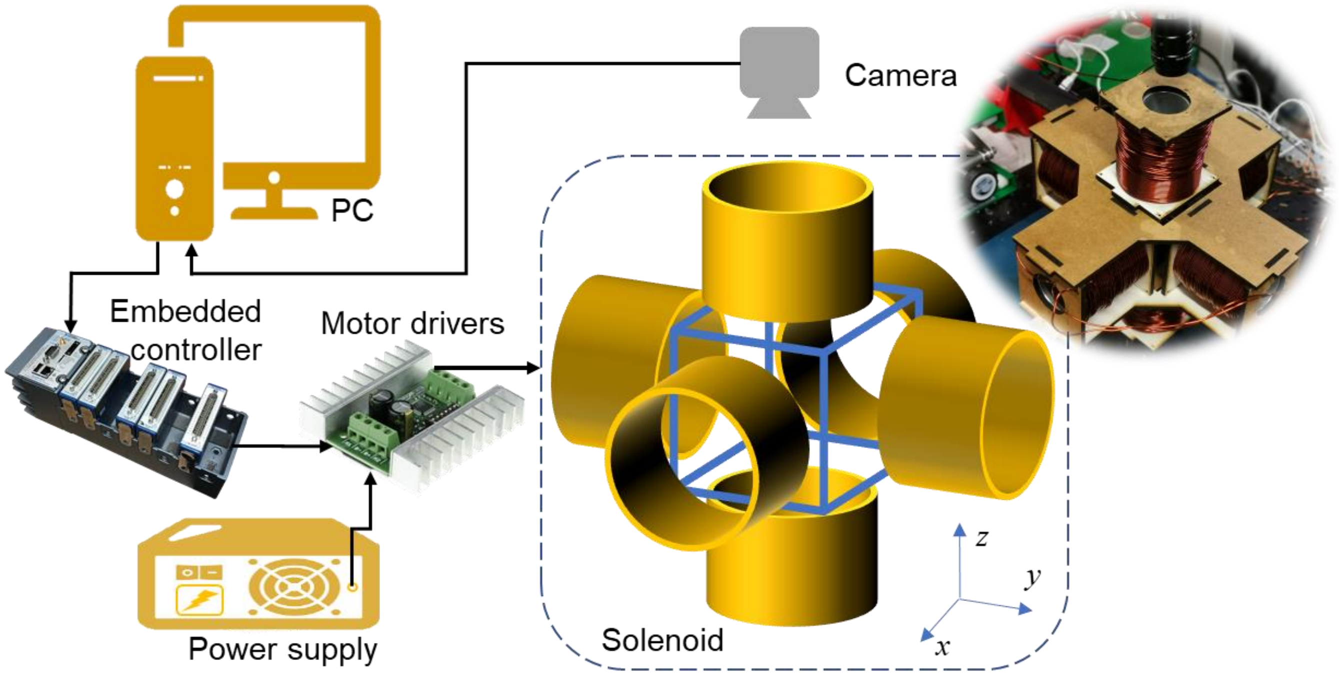

Fig. S3. Electromagnetic actuation system for controlling the soft climbing robot. The customized electromagnetic actuation system consists of a PC as a host controller, a real-time embedded controller (CompactRIO, National Instruments) as a lower controller, an electronic board of six motor drivers (Syren 25, Dimension Engineering), and three pairs of solenoids for generating a uniform magnetic field in the workspace. There are two cameras (Backfly S USB3, Flir System) connected to the PC for visualizing and recording the experiments. The electromagnetic system is able to produce a uniform magnetic field across a cubic area of 2 cm by 2 cm by 2 cm with a magnitude up to 30 mT.

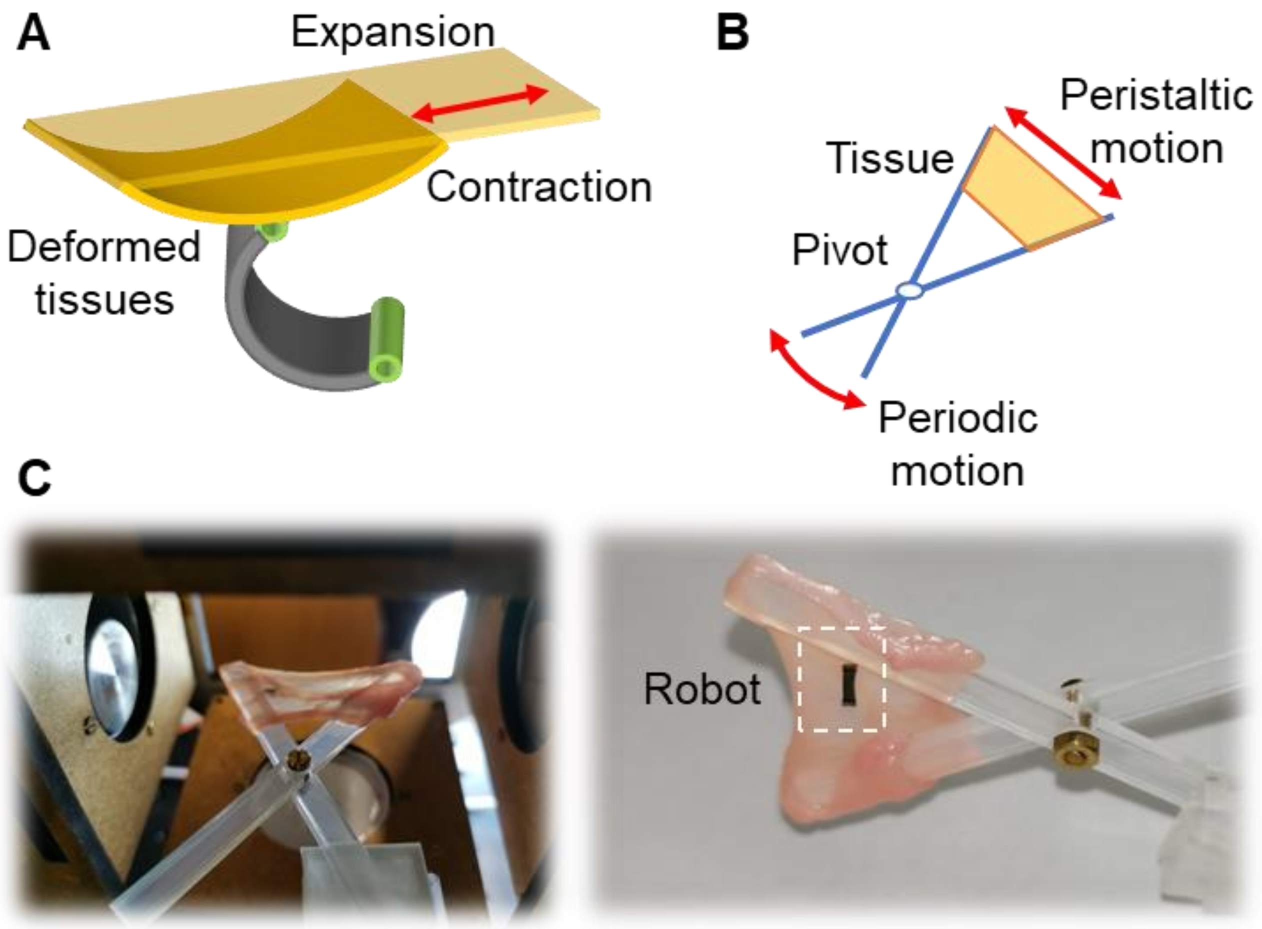

Fig. S4. Experimental setup for testing the ability of the soft climbing robot for withstanding a peristaltic disturbance. A. Schematic of the peristaltic disturbance produced at the tissue substrates. **B.** Illustration of the 'scissor' structure that produces the artificial peristaltic disturbance. Periodic motion was applied manually at approximately 0.5 Hz at the rear side of the structure, yielding peristaltic motion of the tissue at the front side. **C.** Image of the experimental setup. The structure arms were made of PMMA by a laser cutting machine (Epilog Mini 24, Epilog, Inc.) and assembled by a screw and nut at the pivoting point.

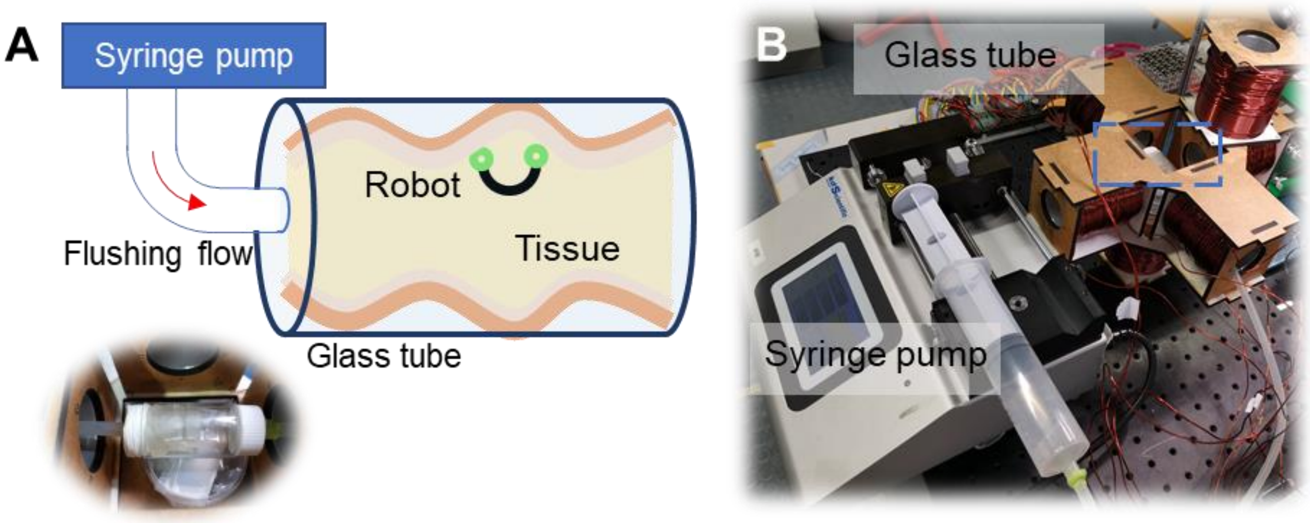

Fig. S5. Experimental setup for testing the ability of the soft climbing robot for withstanding a flushing flow. A. Schematics of the experimental setup. The soft climbing robots with and without a patterned body surface were loaded on a piece of porcine small intestine tissue in a tubular structure. The small intestine tissues attached to the corresponding phantom model was placed in a glass tube with a 25 mm inner diameter, while both ends are sealed and connected to a rubber tube of 7 mm inner diameter. The flushing speeds of water were controlled by a syringe pump. **B.** Image of the experimental setup for testing. The flushing flow was generated and controlled via a syringe pump with a 100 mL syringe. Green plastic spherical particles (200 **µm** in diameter) were mixed with deionized water to help track the speed of the fluid flow. The robots with a plain body and a patterned body were placed on the small intestine tissue axial-symmetrically about the glass tube at similar locations so that both robots would bear the same flushing flow.

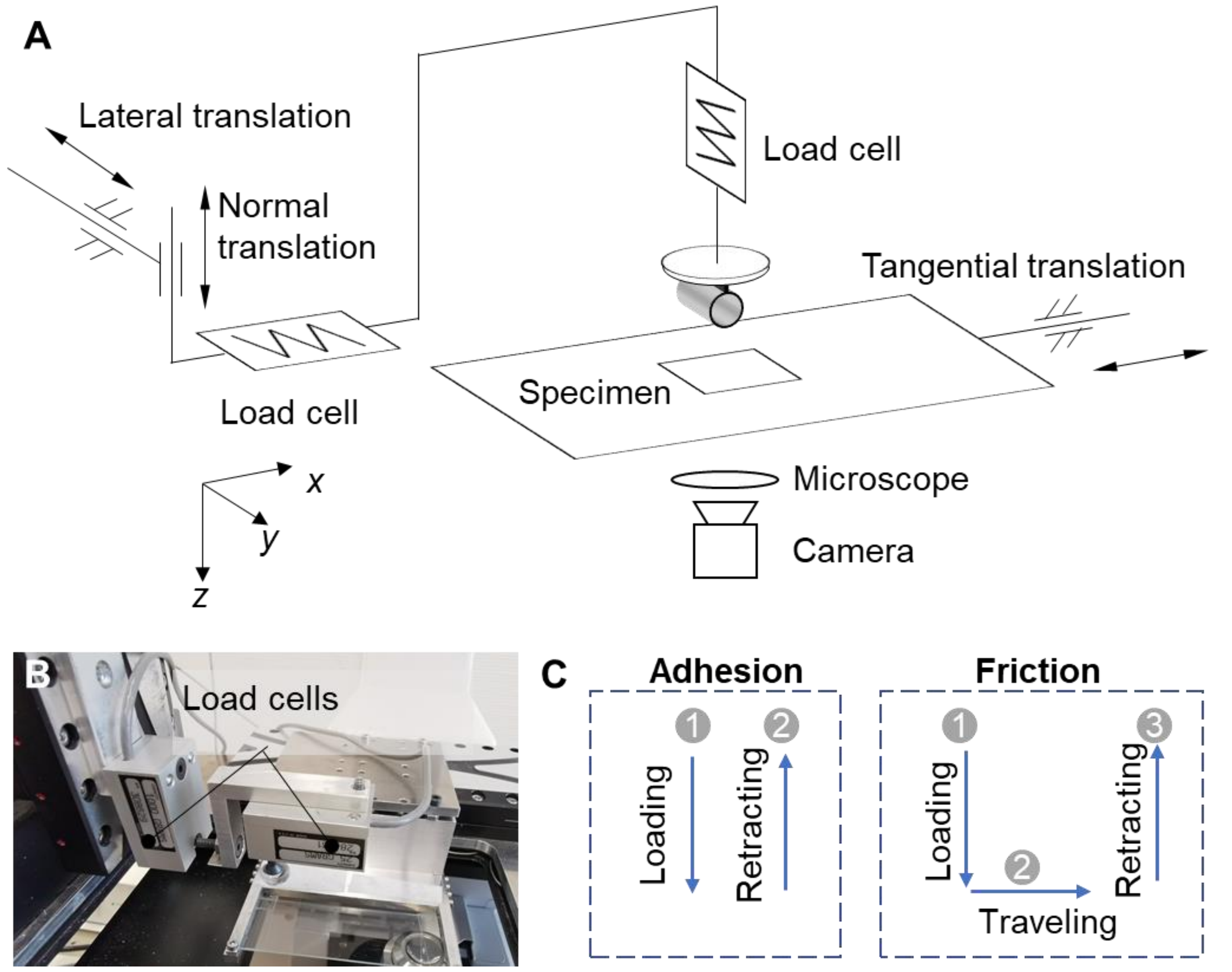

Fig. S6. Customized experimental setup for the adhesion and friction measurements. A. Schematic of the setup. The adhesion and friction were measured using two load cells connected to the sensor probe vertically and horizontally, respectively. The load cells were mounted on a motorized stage that can translates vertically. The specimen was placed on a glass stage mounted on another motorize stage as a tangential translation station. **B.** Image of the experimental setup. **C.** Illustration of the process of the adhesion and friction tests.

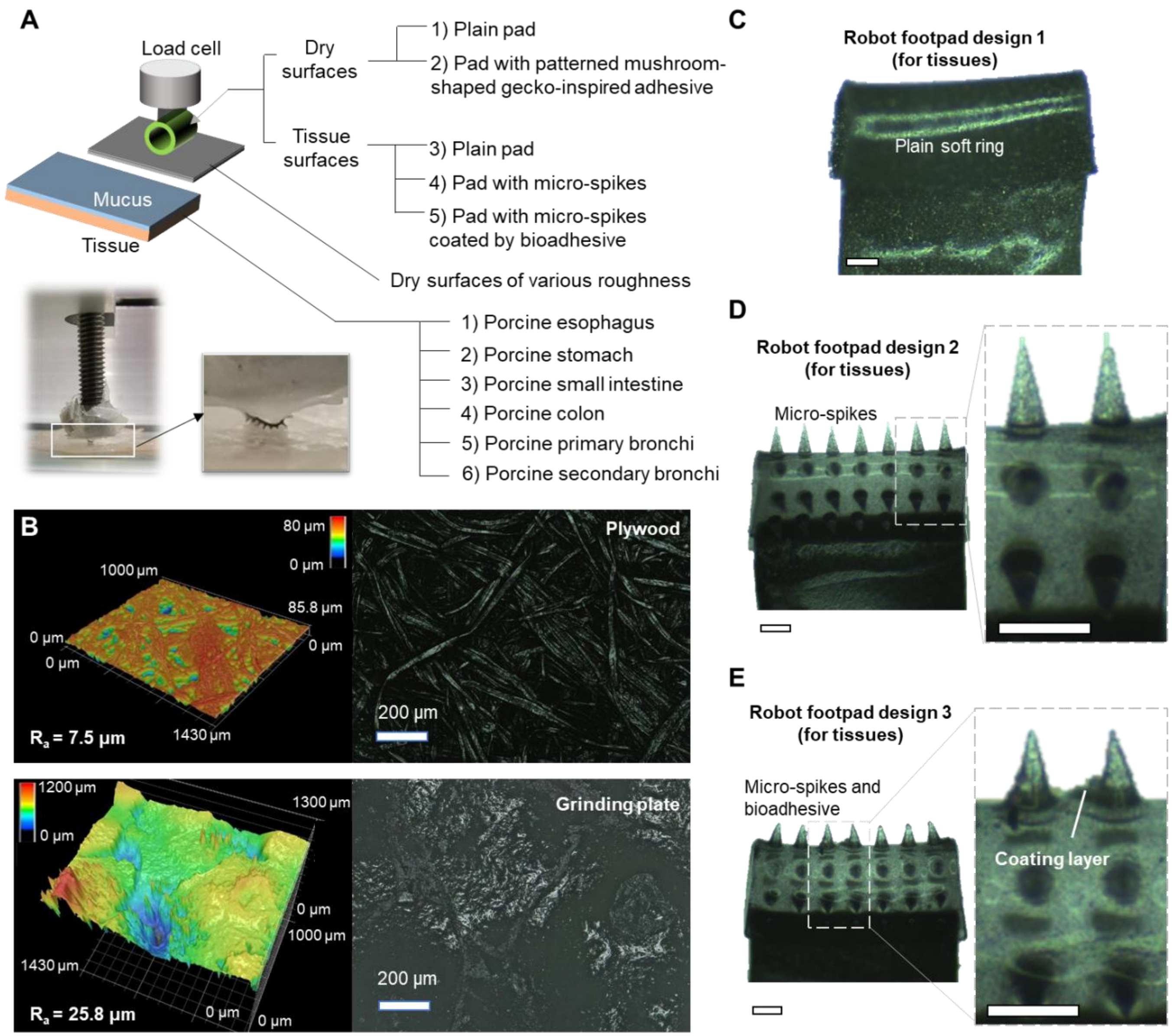

Fig. S7. Illustration of characterizing the adhesion and friction of different robot footpad designs on dry rough surfaces and porcine tissues. A. The configuration of the characterization setup, the robot footpads, and the surfaces. The footpads were prepared as for the robot and attached to the probe. The dry surfaces tested included the glass slide ($\boldsymbol{R_a}$ = 1.8 **µm**), plywood ($\boldsymbol{R_a}$ = 7.5 **µm**), inner surface of a grinding paper roll ($\boldsymbol{R_a}$ = 19.6 **µm**) and the grinding plate ($\boldsymbol{R_a}$ = 25.8 **µm**) and the tissue surfaces tested included the porcine esophagus, stomach, small intestine, colon, primary bronchi, and secondary bronchi. All tissues were freshly prepared for every 10 tests (within 5 minutes) to avoid the dehydration of the tissues. **B.** The laser-optical scanning images of the plywood and the grinding plate surfaces. **C.** The robot footpad Design 1 for tissues with the plain soft ring as the pad. **D.** The robot footpad Design 2 for tissues with micro-spikes on the soft ring. **E.** The robot footpad Design 3 for tissues with bio-adhesive coated micro-spikes on the soft ring. The irregular glint on the pad compared with that in **C** indicates the coating layer of the hydrogel and bio-adhesive. Scale bars: 200 **µm**.

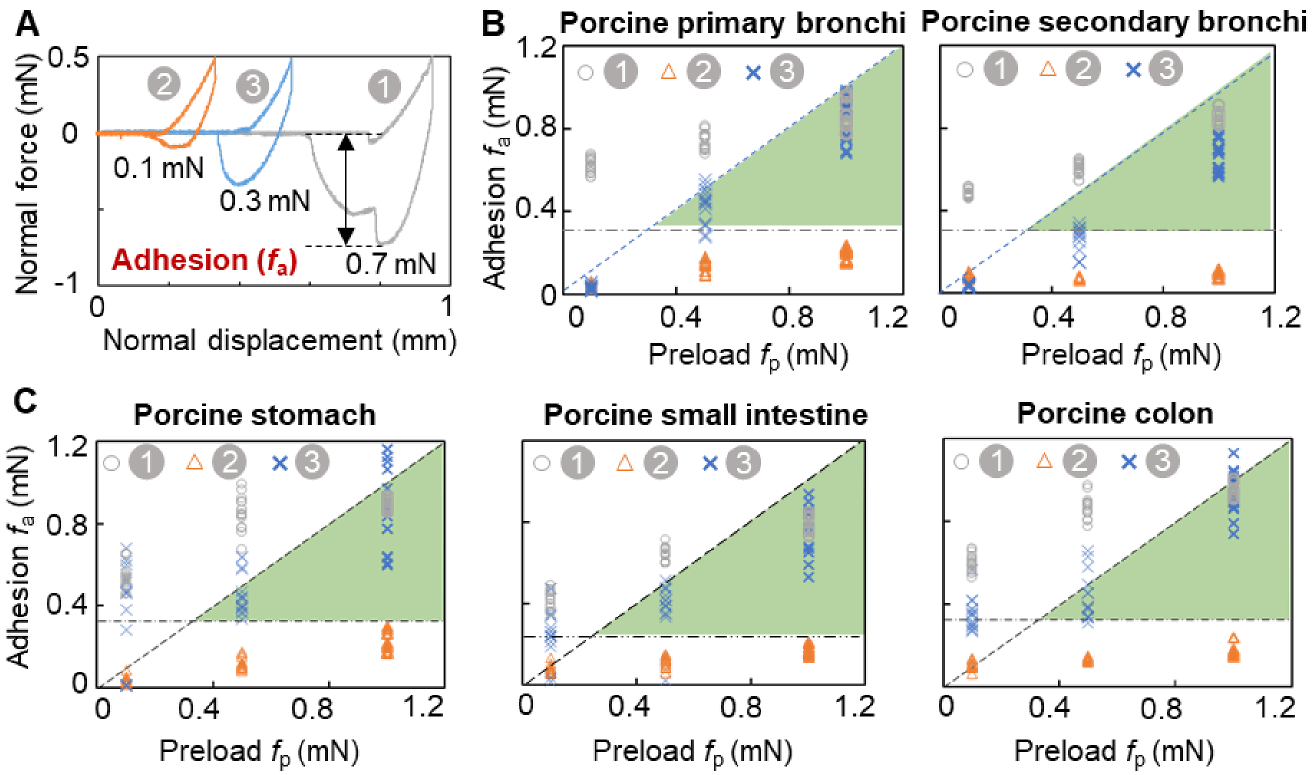

Fig. S8. Characterization of the adhesion of three footpad designs on porcine tissues. A. Normal force curve measured for footpads of the three designs for tissues on a porcine esophagus tissue surface ex vivo under a preload of 0.5 mN and a contact time of 5 seconds. The adhesion $\boldsymbol{f_a}$ is quantified using the maximum pull-off force (negative normal force) as denoted in the plot. The footpad in Design 1 shows the adhesion around 0.74 mN larger than the preload applied 0.5 mN, which makes peeling process more difficult than the loading process. The footpad in Design 2 shows the adhesion around 0.11 mN which is not enough for anchoring of the robot on the inverted tissue surface. The unqiue footpad in Design 3 shows adhesion that is within a proper range. **B.** Characterization of adhesion between the three footpad designs and the tissue surfaces on porcine respiratory tract. The adhesion $\boldsymbol{f_a}$ is plotted as a function of the preload $\boldsymbol{f_p}$= 0.1, 0.5, 1 mN and the contact time $\boldsymbol{\Delta t}$ = 1, 5 ,10 s, respectively. The tissues used here were from fresh porcine primary bronchi and secondary bronchi. The green region denotes a feasible region where the robot pad could be peeled off using similar forces after being loaded to the substrate. **C.** Characterization of the adhesion between the three pad designs and the tissue surfaces on porcine GI tract. The tissues used here were from a fresh porcine stomach, small intestine and colon. The experiment conditions and data processing are the same as **B.**

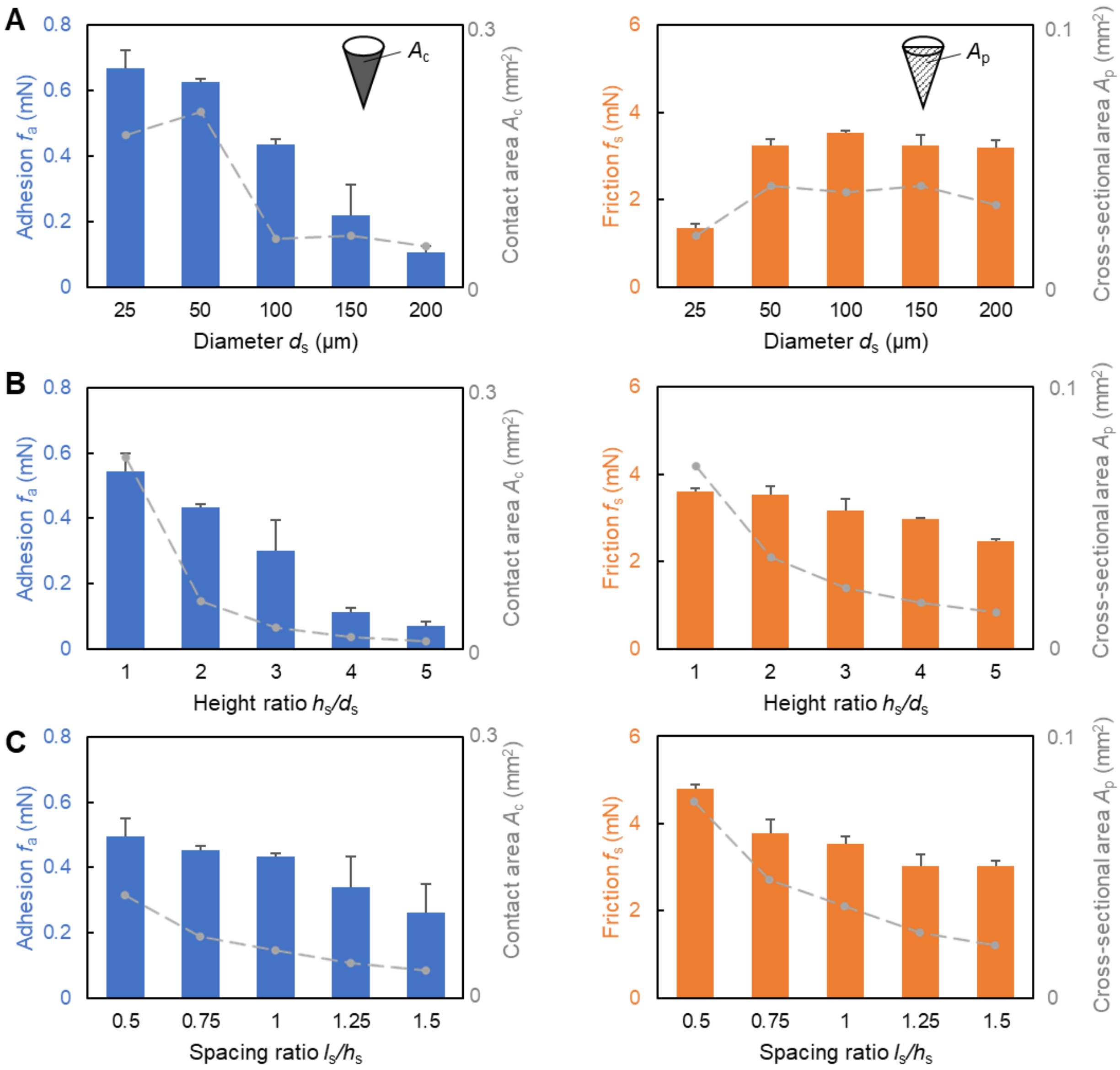

Fig. S9. Adhesion and friction of ring pads with micro-spikes on porcine small intestine tissues as a function of various geometric parameters. A. Adhesion and friction measured as a function of the diameter of spikes, $\boldsymbol{d_s}$, while keeping the height ratio, $\boldsymbol{h_s/d_s}$, and spacing ratio, $\boldsymbol{l_s/h_s}$, as 2 and 1 respectively. **B.** Adhesion and friction measured as a function of the height ratio of spikes, $\boldsymbol{h_s/d_s}$, while keeping the diameter, $\boldsymbol{d_s}$, and spacing ratio, $\boldsymbol{l_s/h_s}$, as 200 μm and 1 respectively. **C.** Adhesion and friction measured as a function of the spacing ratio of spikes, $\boldsymbol{l_s/h_s}$, while keeping the diameter, $\boldsymbol{d_s}$, and height ratio, $\boldsymbol{h_s/d_s}$, as 200 μ**m** and 2 respectively. Error bars represent the standard deviation of n = 5 measurements. Each test contains at least five repetitions. In all experiments, adhesion test conditions: preload 0.5 mN, contact time 5 seconds. The adhesion measured is highly correlated with the real contact area of the micro-spike design, namely the area of the outer surface of the spikes, while the friction measured is related to the cross-sectional area, which is the projected area in the same direction as the tangential motion.

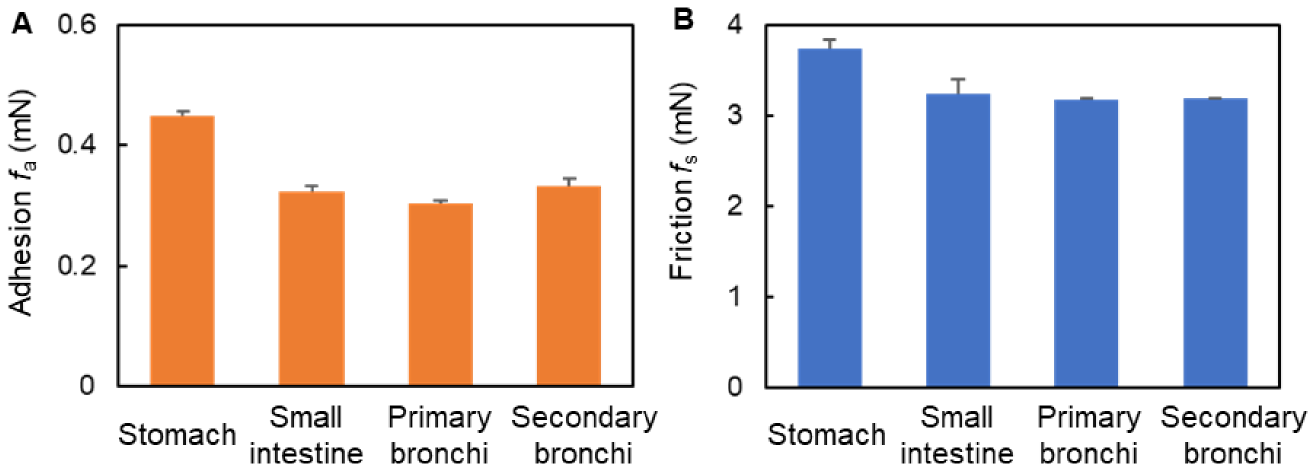

Fig. S10. Characterization of the adhesion and friction of the robot footpad with hydrogel-coated hollow spikes. A. Adhesion f_a of the bio-adhesive coated hollow micro-spike pad loaded by drug analog on porcine tissues. Adhesion test conditions: preload 0.5 mN, contact time 5 sec. **B.** Friction f_s of the bio-adhesive coated hollow micro-spike pad loaded by drug analog on porcine tissues. Tests were conducted on porcine tissues, including the stomach and small intestine for GI tract and the primary bronchi and the secondary bronchi for respiratory tract. Error bars represent the standard deviation for $n = 5$ measurements.

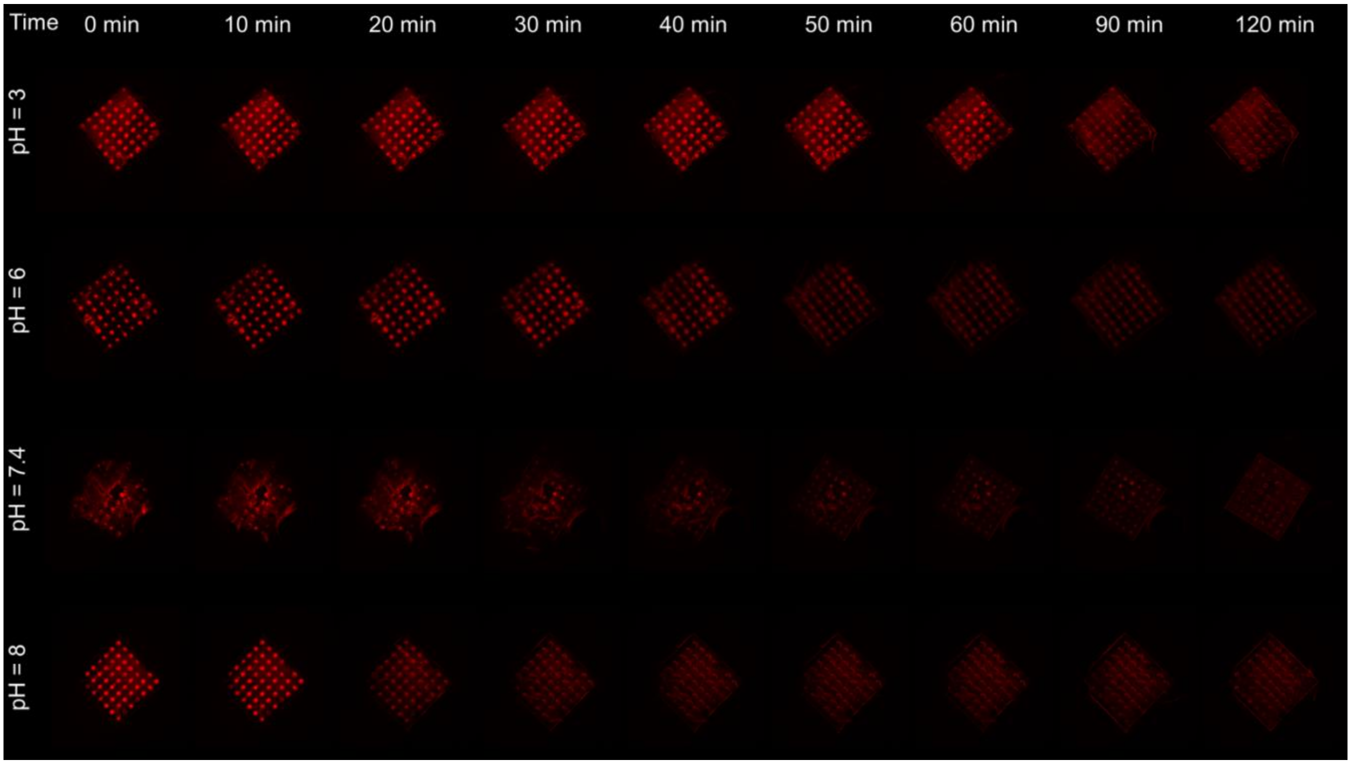

Fig. S11. Experimental fluorescence images of the robot footpads in standard PBS buffers of various pH values over time. The footpads were fully merged in various standard buffers with a pH value of 3, 6, 7.4 and 8. The fluorescence intensities (bandpass emission: 670 nm) of the footpads were measured using a fluorescence microscope (Nikon Inverted Microscope Eclipse Ti-E, Nikon, Inc). The sampling rate is one image per minute and the measurement lasts for 3 hours for each pad.

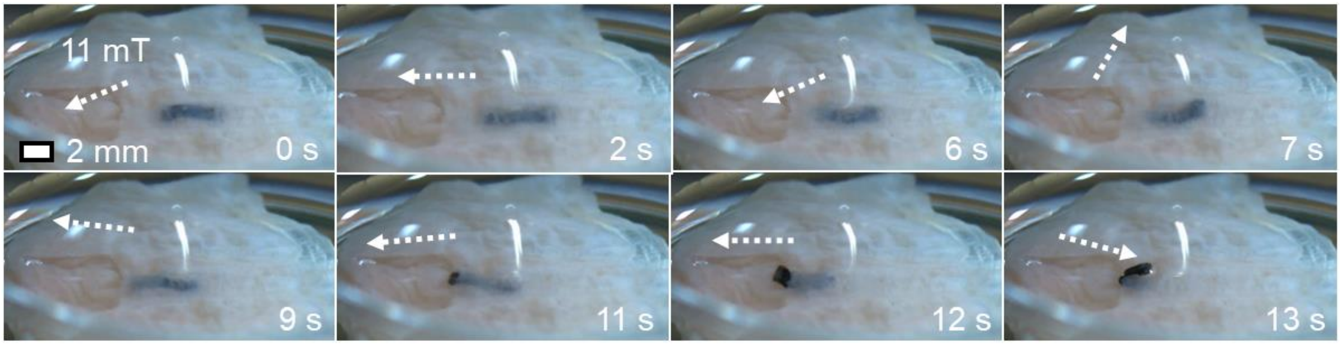

Fig. S12. Sequential images of the soft climbing robots crawling in collapsed porcine small intestine tissue surfaces ex vivo. The proposed soft climbing robot navigated between collapsed tissues using a crawling locomotion. The micro-spikes on the robot footpad enhance the tissue friction. A rotating magnetic field of 11 mT was applied to induce the crawling locomotion.

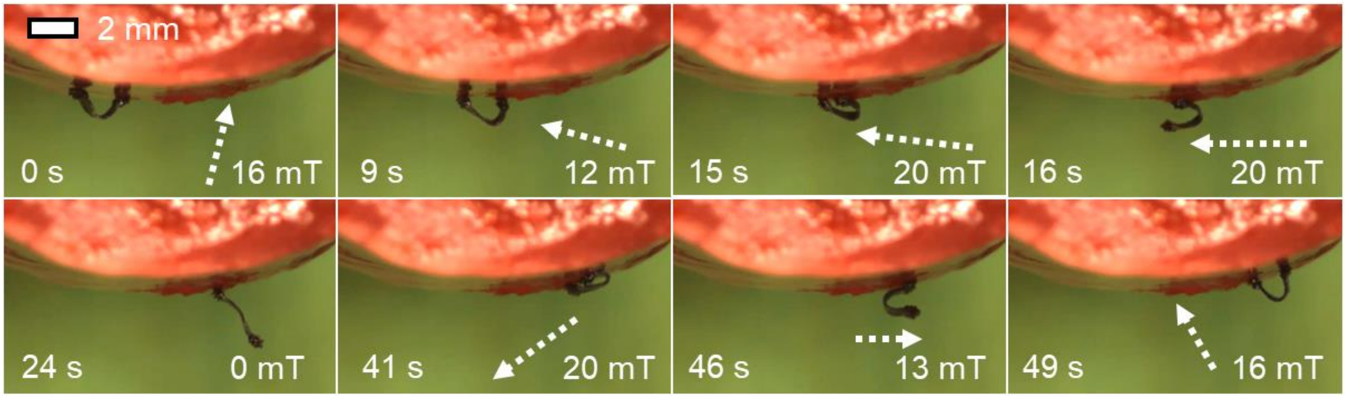

Fig. S13. Sequential images of the soft climbing robots climbing on a porcine heart outer layer tissue surface ex vivo. The proposed soft climbing robot with the adhesive pads designed especially for the tissues covered by mucus can also climb on the porcine heart outer layer tissue surface. A further design based on targeted tissues surface properties can make it able to climb on other different tissues.

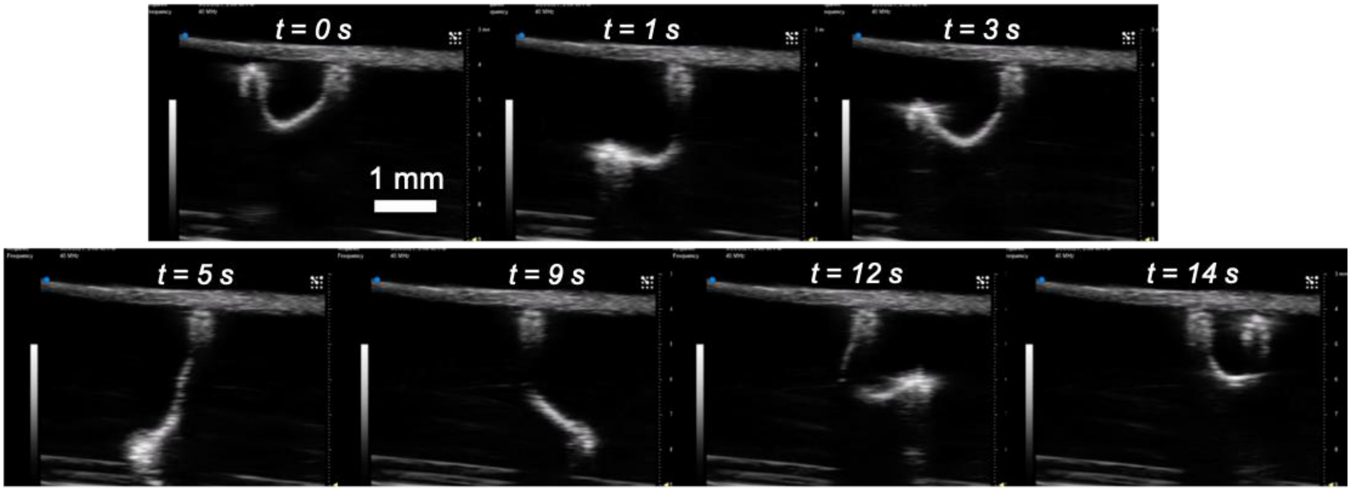

Fig. S14. Sequential images of the soft climbing robots climbing on a porcine small intestine tissue ex vivo with ultrasound imaging guidance. The tissue was merged in water for a better contrast of the ultrasound imaging. The ultrasound imaging machine is Vevo 2100 from FUJFILM VisualSonics, Inc.

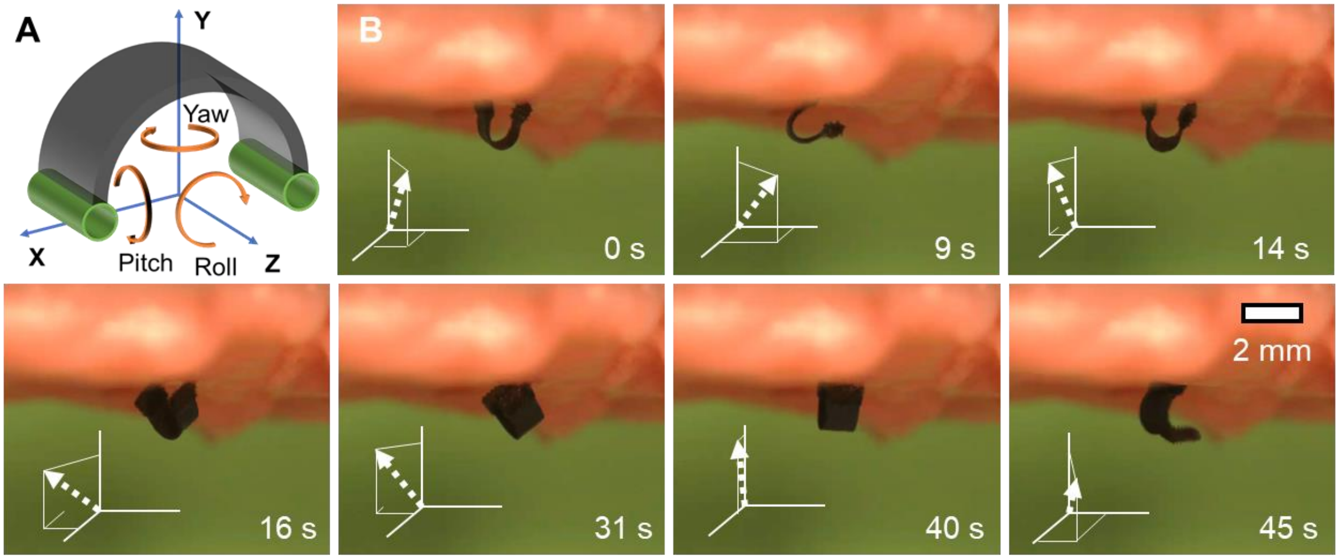

Fig. S15. Steering of the soft robot during climbing. A. Schematic of the 3D body rotation via roll, pitch, and yaw. **B.** Sequential video snapshots of the soft climbing robots steering invertedly. The robot was steered to rotate by 90° in the yaw direction on a porcine small intestine tissue ex vivo under a magnetic field with an out-of-plane component. **B** field: 12 mT with its orientation indicated by white arrows.

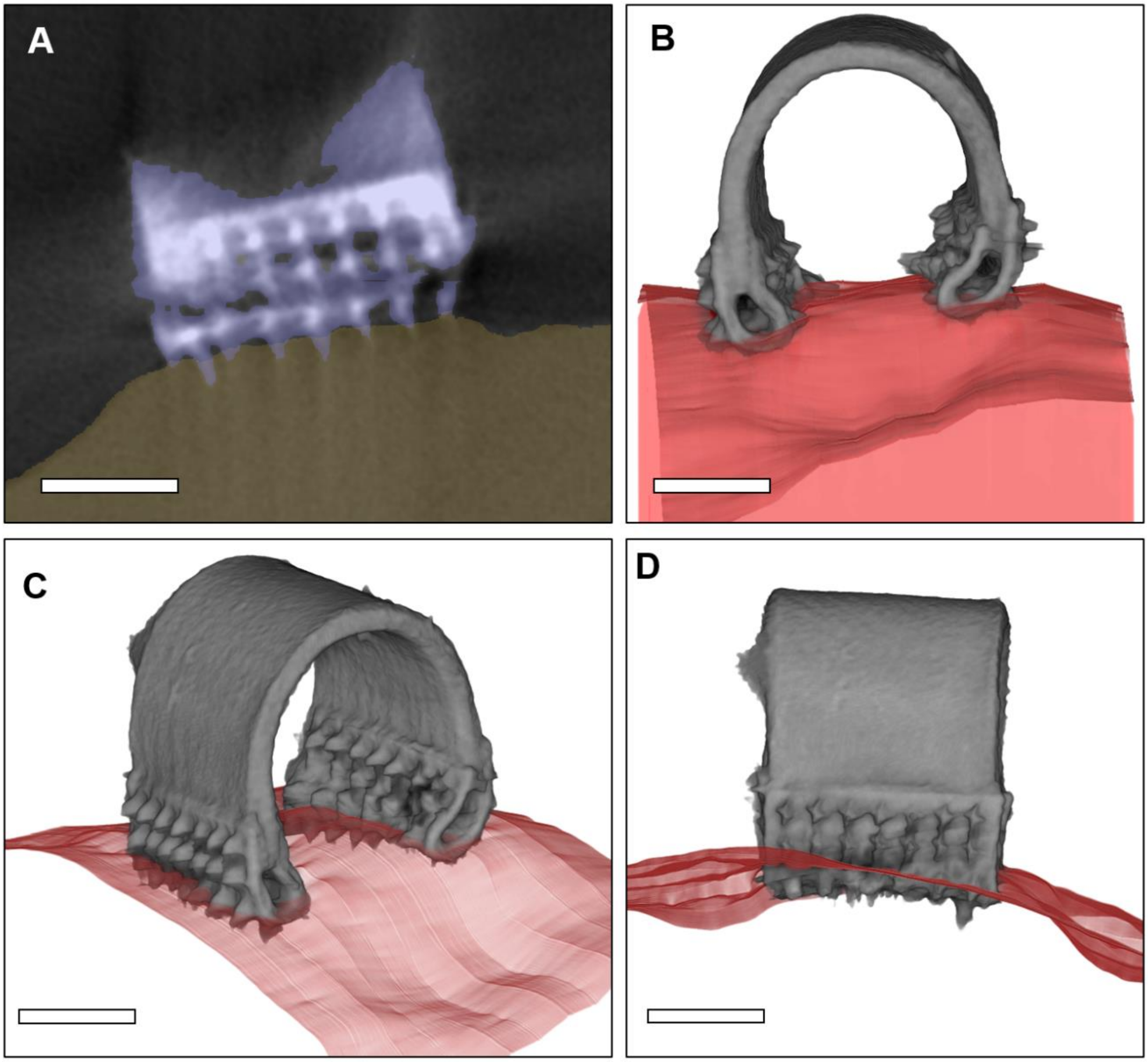

Fig. S16. 3D reconstruction of the robot footpads penetrating mucus layers based on X-ray computational tomography imaging. A. X-ray image of the soft robot penetrating the mucus layers. (**B-D**) 3D reconstruction images in a (**B**) front view, (**C**) perspective view, and (**D**) side view of the robot pads penetrating the mucus layer of porcine small intestine tissues. Scale bars, 500 **μm**.

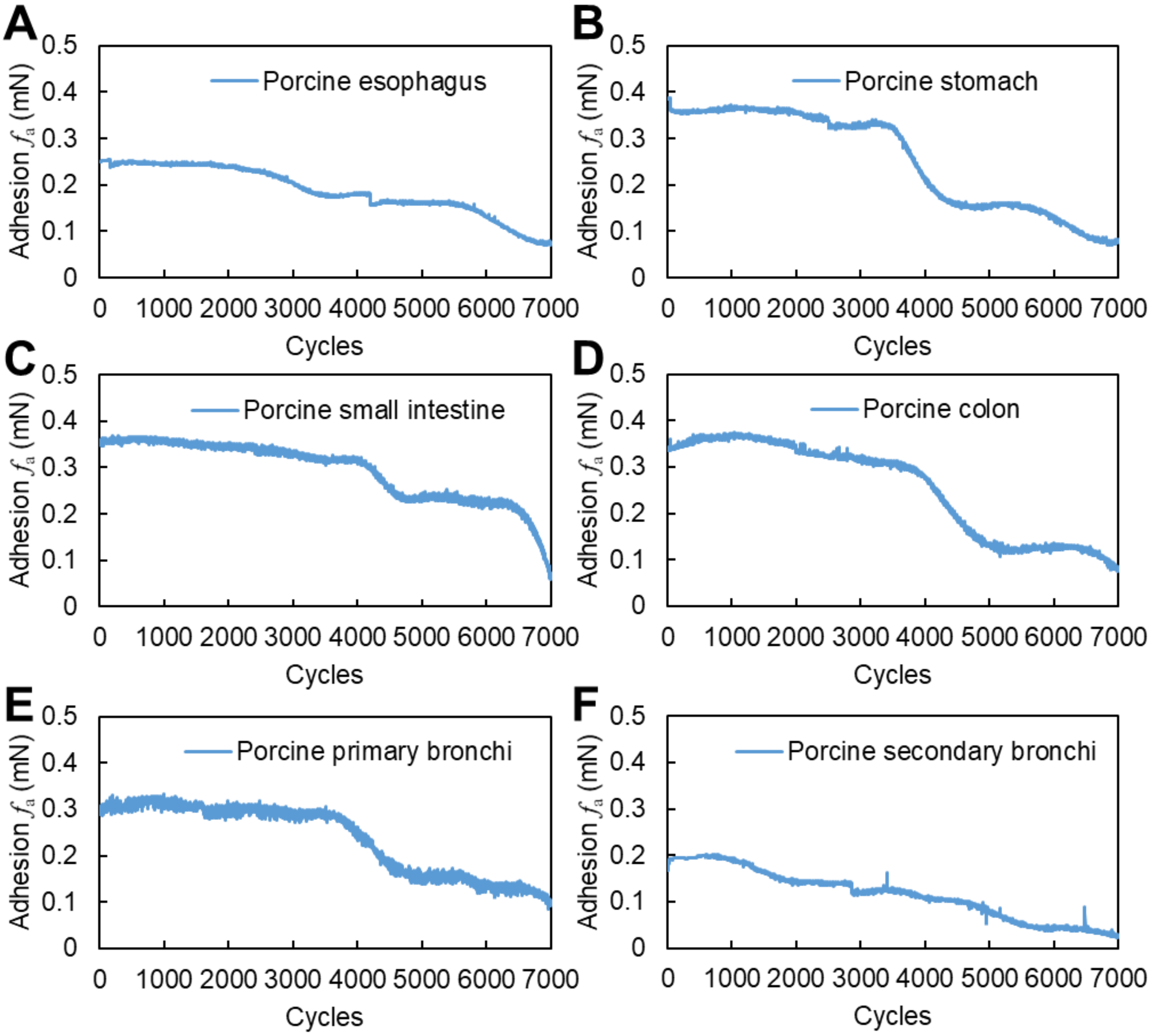

Fig. S17. Measured adhesion of the robot footpad in design 3 on porcine tissues as a function of the loading-peeling cycle number. The measurement conditions include preload: 0.5 mN, contact time: 1 second, relative humidity: 95%, and temperature: 37 °C.

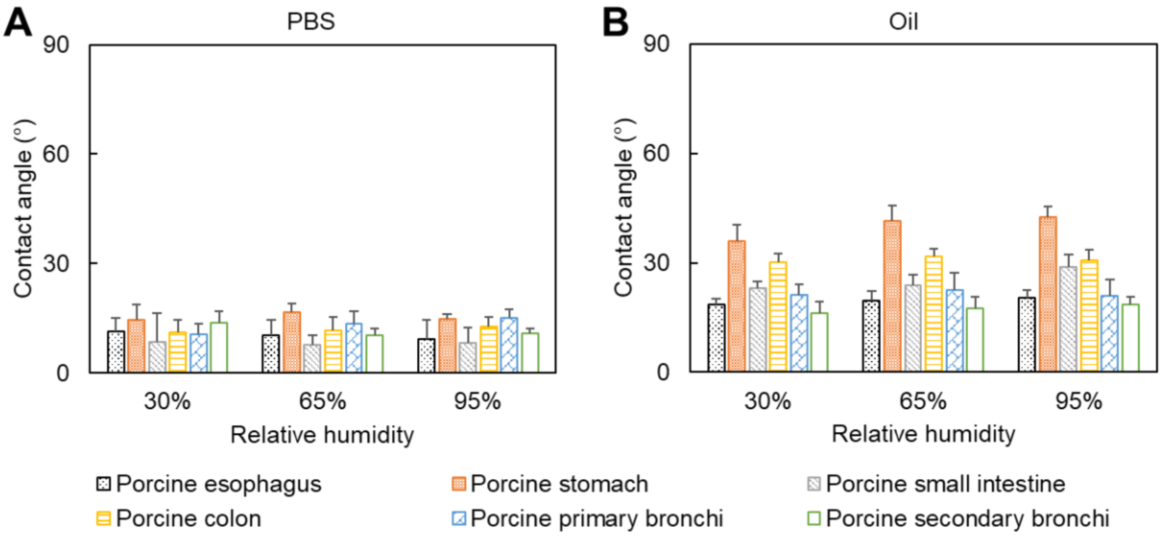

Fig. S18. Surface contact angles of fresh porcine tissues as a function of the relative humidity conditions. A. Contact angle of the standard Phosphate-Buffered Saline (PBS) solution on porcine tissue surfaces. **B**. Contact angles of vegetable oil (Rape seed, K-class, Kaufland AG) on porcine tissue surfaces. Temperature: 37°C. Error bars represent the standard deviation of n = 5 measurements.

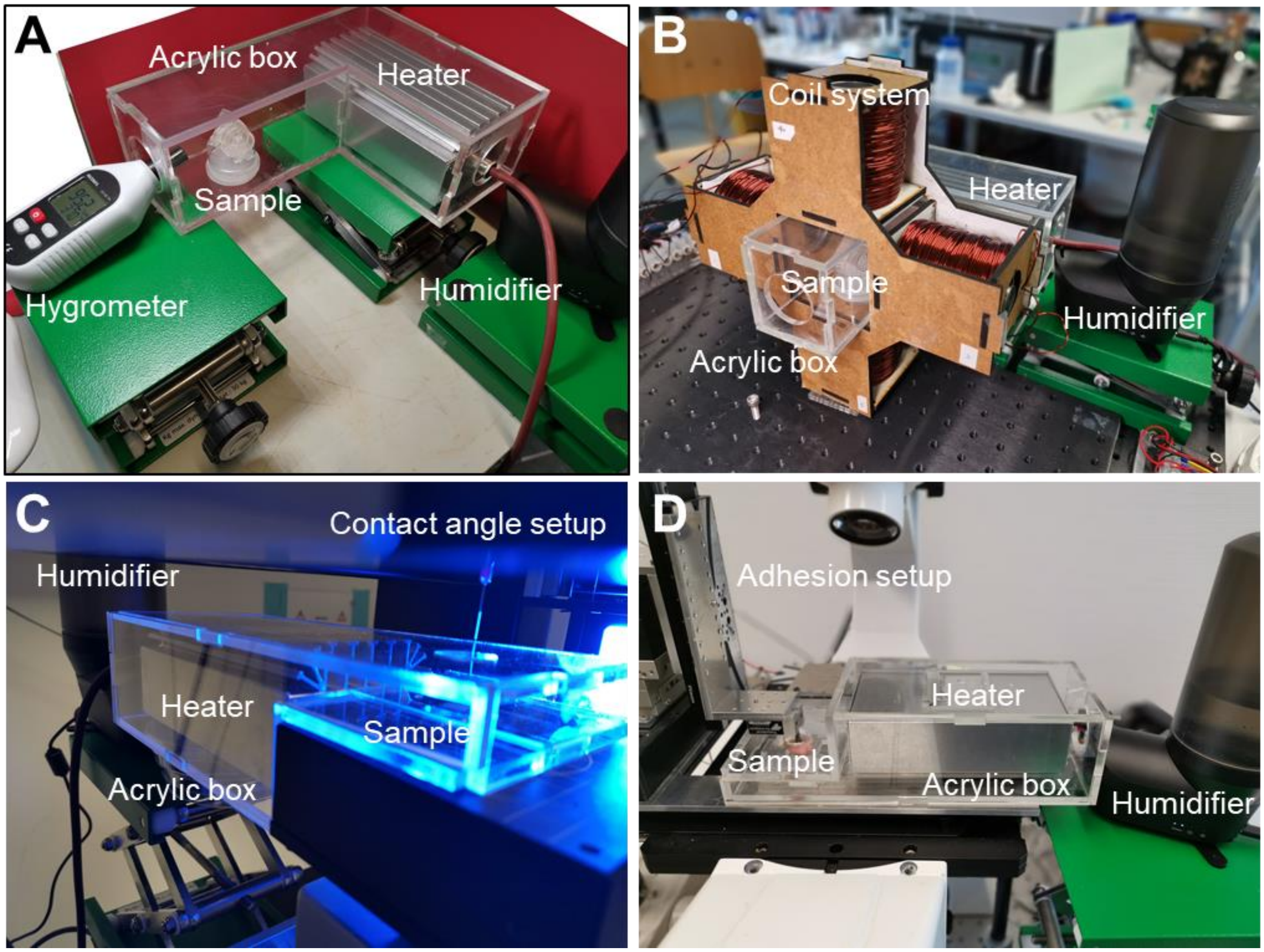

Fig. S19. Customized experimental setups integrating the module for controlling the relative humidity and temperature. A. Image of the experimental setup to control the relative humidity and temperature of a workspace. The samples were placed in a 1.92-liter L-shaped acrylic box with holes of 4 cm in diameter at both ends of the box for ventilation. A heater (LM-Standard, RO/SE Blechverarbeitung GmbH & Co. KG) was placed inside the box for controlling the temperature inside the box. An air humidifier (Emma, Stadler Form GmbH) was placed right at the far side hole to the sample for increasing the relative humidity inside the box. A hygrometer (VOLTCRAFT HY-10, Voltcraft Engineers Private Limited) was used at the near side hole to the sample for monitoring the relative humidity and the temperature inside the box. **B.** Image of the electromagnetic coils with the experimental module for controlling humidity and temperature. **C.** Image of the Droplet Shape Analyzer (DSA100, KRACESS GmbH) integrating the module with humidity and temperature control for the contact angle measurements. **D.** The experimental setup for the adhesion measurements with humidity and temperature control. The conditions are similar to the intra-abdominal humidity of the human body from 30% to 95% (*63*). In all experiments, the tissues were kept under the set conditions for at least 20 minutes before the tests.

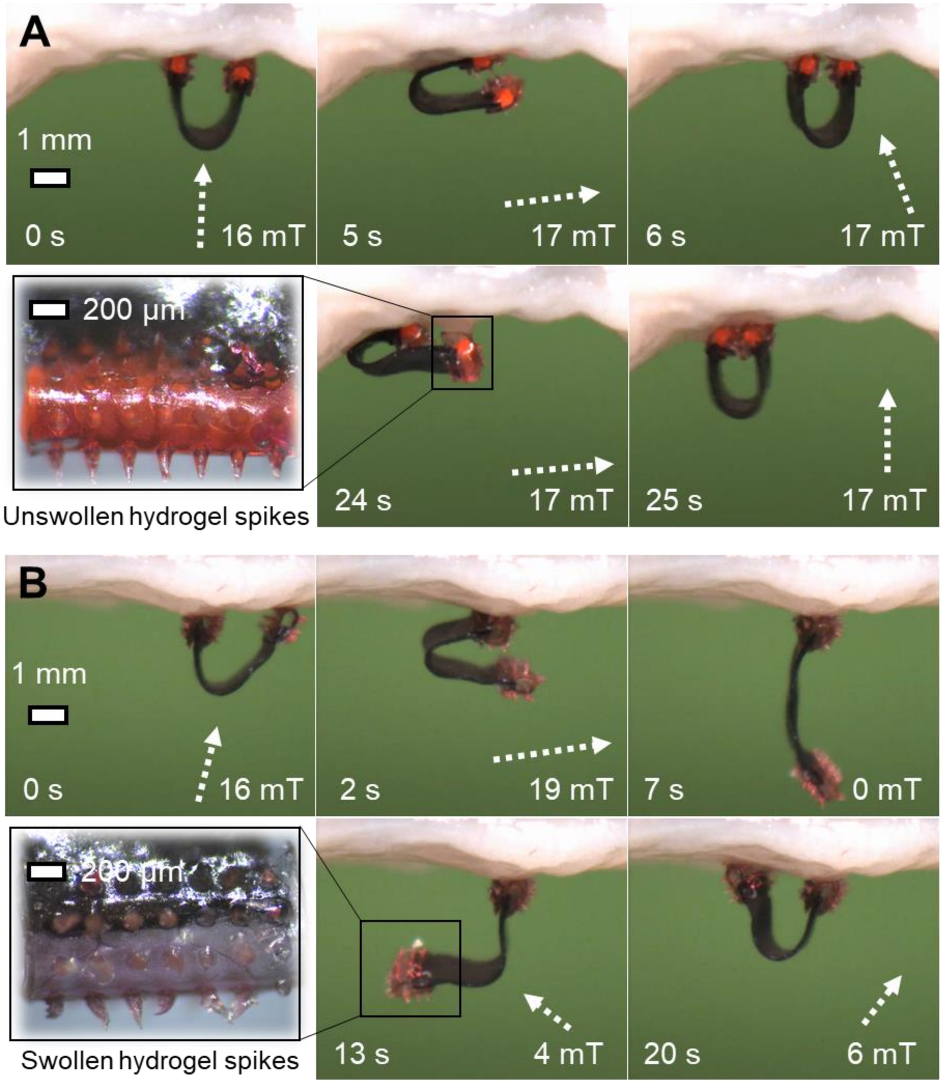

Fig. S20. Comparison of unswollen and swollen hydrogel spikes for robot climbing tissue surfaces ex vivo. A. Sequential images of a robot climbing a porcine small intestine tissue using freshly prepared robot footpads with unswollen hydrogel spikes. **B.** Sequential images of a robot climbing a porcine small intestine tissue using robot footpads with swollen hydrogel spikes. The robot footpads had been in contact with the porcine small intestine mucus layer for 2 hours before the climbing tests.

Supplementary Note 1. **Force analysis in the peeling-and-loading mechanism.**

We have the following force balancing equation for the whole robot (**fig. S21A**) with a pinned-pined boundary condition, which is given by

$$\mathbf{0} = \sum \mathbf{F} = \mathbf{G} + \mathbf{F}_1 + \mathbf{F}_2, \quad (1)$$

where $\mathbf{F}_1$ and $\mathbf{F}_2$ are the contact forces applied by the substrate surface on the robot left and right footpads, respectively. We also have the force balancing equations for the two robot footpads (**fig. S21B**), given by

$$\mathbf{F}_{\text{loading}} + \mathbf{F}_1 = \mathbf{0}, \quad (2)$$

$$\mathbf{F}_{\text{peeling}} + \mathbf{F}_2 = \mathbf{0}. \quad (3)$$

Meanwhile, we also have the moment balancing equations about point a and point b for the whole robot (**fig. S21C**), which is given by,

$$\mathbf{0} = \sum \mathbf{M}_{\text{a}} = \boldsymbol{\tau}_{\text{net}} + \mathbf{r}_{\text{ac}} \times \mathbf{G} + \mathbf{r}_{\text{ab}} \times \mathbf{F}_2, \quad (4)$$

$$\mathbf{0} = \sum \mathbf{M}_{\text{b}} = \boldsymbol{\tau}_{\text{net}} + \mathbf{r}_{\text{bc}} \times \mathbf{G} + \mathbf{r}_{\text{ba}} \times \mathbf{F}_1, \quad (5)$$

where r_{ab} and r_{ba} represent the moment arms of $\mathbf{F}_1$ and $\mathbf{F}_2$ about point a and point b, respectively. r_{ac} and r_{bc} represent the moment arms of $\mathbf{G}$ about point a and point b, respectively. $\boldsymbol{\tau}_{\text{net}}$ is the net external magnetic torque given by $\boldsymbol{\tau}_{\text{net}} = \mathbf{M}_{\text{net}} \times \mathbf{B}(t)$, where $\mathbf{M}_{\text{net}}$ is the net magnetic moment given by $\mathbf{M}_{\text{net}} = \int_0^L \mathbf{R}[\theta(s)] \cdot \mathbf{M}(s)\, wt ds$. The internal force and bending moment do not contribute to the whole-body torque. By solving Equations (1)-(5), the normal component of the peeling and loading forces could be estimated from the shape of the deformed robot body and the external magnetic fields. The tangential components of the peeling and loading forces are more complex and could be estimated using the bending moment of the deformed robot soft body described in (*6*, *11*). We will explore these theoretical models in more details in the future work.

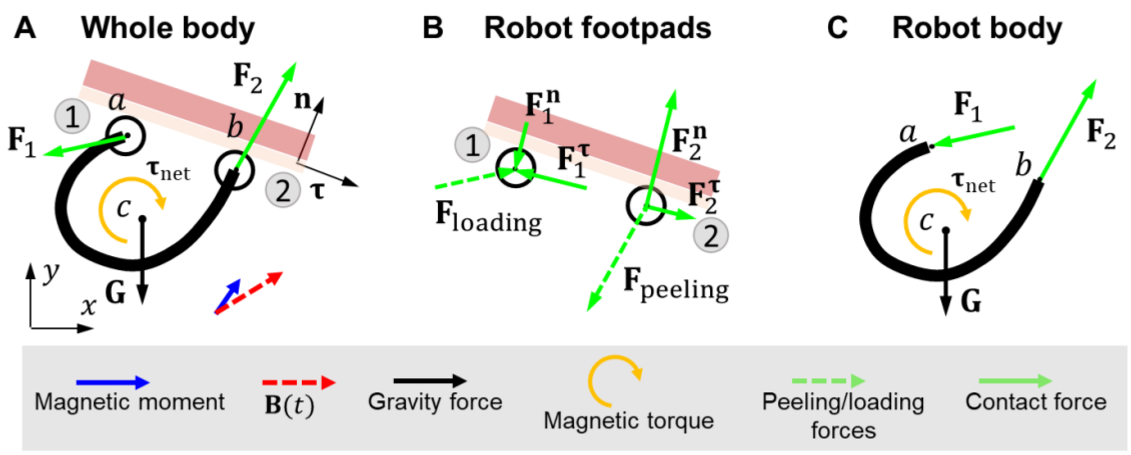

Fig. S21. The force and moment analysis of the robot footpads and body when adhering to an inverted and inclined surface. A. Force and moment diagram of the whole robot**. B.** Free-body force diagram of the robot footpads**. C.** Force and moment diagram of the robot body. Point a and point b represent the contact points of the left and right footpads on the substrate surface. The net magnetic torque τ_{net}, together with the gravity **G**, induces the loading ($\mathbf{F}_{\text{loading}}$) and peeling ($\mathbf{F}_{\text{peeling}}$) forces on the robot footpads. $\mathbf{F}_1$ and $\mathbf{F}_2$ are the contact forces consisting of the adhesion, friction and supporting forces, which

are applied by the substrate surface on the left and right robot footpads, respectively. The superscripts $\boldsymbol{\tau}$ and $\mathbf{n}$ denote the tangential and normal components of the contact forces.

Supplementary Movies

Movie S1. The climbing mechanism of the soft robot.
This video presents the mechanism of controlling the robot soft-body deformation and the loading-and-peeling behavior by varying external magnetic field, as well as the experimental and simulated walking-based climbing locomotion and the tumbling-based climbing locomotion.

Movie S2. Climbing on 3D dry and rough surfaces.
This video shows that the robot with footpads integrating dry adhesives climb on different 3D dry and rough surfaces. The robot is also shown to climb in torturous and confined tubular structures.

Movie S3. The unique design of the robot footpads for tissue adhesion and friction.
This video presents the comparison of three types of robot footpad designs for tissues. The three designs are all made of PDMS, but the first design has only a plain robot pad (Design 1) without any micro-structures or coated bioadhesives, the second design (Design 2) has only micro-structures, while the third design (Design 3) has micro-structures that are sequentially coated by hydrogel and bioadhesives. The climbing performance on porcine GI tract tissues are compared for robots with the three different robot footpad designs.

Movie S4. Climbing on versatile tissue surfaces.
This video shows that the soft climbing robot with the unique robot footpad design climbs on various porcine tissues in the GI tract and the respiratory tract.

Movie S5. The function of carrying cargos and traversing complex terrains with multi-modal locomotion.
This video presents that the soft climbing robots carry various payloads with large weights and volumes. It also shows that the robot carries a soft capsule for liquid drug delivery, traverse complex terrains with multi-modal locomotion and deliver the liquid drug on-demand at the target locations.

Movie S6. The function of pH-responsive drug delivery by penetrating mucus layers.
This video presents the process of the pH-responsive drug release process of the robot footpad under a fluorescence microscope, the various climbing locomotion on tissue surfaces of the soft robot with hydrogel micro-spikes while carrying drugs, and the long-term retention ability of the robot on tissue surfaces while withstanding fluid flows.

Movie S7. Climbing porcine tissue surfaces with controlled humidity and temperature.
This video presents that the robot climbs porcine primary bronchi, esophagus and colon ex vivo under controlled environmental conditions (temperature and relative humidity) similar to that in the human body. The conditions of 37°C and 65% relative humidity were used in the porcine primary bronchi, esophagus and colon ex vivo. The conditions of 37°C and 95% relative humidity were used in the porcine secondary bronchi, stomach and small intestine ex vivo.

REFERENCES AND NOTES

1. S. I. Rich, R. J. Wood, C. Majidi, Untethered soft robotics. *Nat. Electron.* **1**, 102–112 (2018).

2. Z. Zhakypov, K. Mori, K. Hosoda, J. Paik, Designing minimal and scalable insect-inspired multi-locomotion millirobots. *Nature* **571**, 381–386 (2019).

3. B. J. Nelson, I. K. Kaliakatsos, J. J. Abbott, Microrobots for minimally invasive medicine. *Annu. Rev. Biomed. Eng.* **12**, 55–85 (2010).

4. M. Sitti, Miniature soft robots—Road to the clinic. *Nat. Rev. Mater.* **3**, 74–75 (2018).

5. Y. Kim, H. Yuk, R. Zhao, S. A. Chester, X. Zhao, Printing ferromagnetic domains for untethered fast-transforming soft materials. *Nature* **558**, 274–279 (2018).

6. G. Z. Lum, Z. Ye, X. Dong, H. Marvi, O. Erin, W. Hu, M. Sitti, Shape-programmable magnetic soft matter. *Proc. Natl. Acad. Sci. U.S.A.* **113**, E6007–E6015 (2016).

7. W. Hu, G. Z. Lum, M. Mastrangeli, M. Sitti, Small-scale soft-bodied robot with multimodal locomotion. *Nature* **554**, 81–85 (2018).

8. H. Lu, M. Zhang, Y. Yang, Q. Huang, T. Fukuda, Z. Wang, Y. Shen, A bioinspired multilegged soft millirobot that functions in both dry and wet conditions. *Nat. Commun.* **9**, 1–7 (2018).

9. H.-W. Huang, F. E. Uslu, P. Katsamba, E. Lauga, M. S. Sakar, B. J. Nelson, Adaptive locomotion of artificial microswimmers. *Sci. Adv.* **5**, eaau1532 (2019).

10. Z. Ren, W. Hu, X. Dong, M. Sitti, Multi-functional soft-bodied jellyfish-like swimming. *Nat. Commun.* **10**, 2703 (2019).

11. X. Dong, G. Z. Lum, W. Hu, R. Zhang, Z. Ren, P. R. Onck, M. Sitti, Bioinspired cilia arrays with programmable nonreciprocal motion and metachronal coordination. *Sci. Adv.* **6**, eabc9323 (2020).

12. B. Wang, K. F. Chan, K. Yuan, Q. Wang, X. Xia, L. Yang, H. Ko, Y.-X. J. Wang, J. J. Y. Sung, P. W. Y. Chiu, Endoscopy-assisted magnetic navigation of biohybrid soft microrobots with rapid endoluminal delivery and imaging. *Sci. Robot.* **6**, eabd2813 (2021).

13. H. Zhang, Z. Li, C. Gao, X. Fan, Y. Pang, T. Li, Z. Wu, H. Xie, Q. He, Dual-responsive biohybrid neutrobots for active target delivery. *Sci. Robot.* **6**, eaaz9519 (2021).

14. A. Ghosh, L. Li, L. Xu, R. P. Dash, N. Gupta, J. Lam, Q. Jin, V. Akshintala, G. Pahapale, W. Liu, Gastrointestinal-resident, shape-changing microdevices extend drug release in vivo. *Sci. Adv.* **6**, eabb4133 (2020).

15. M. M. Mau, S. Sarker, B. Terry, Ingestible devices for long-term gastrointestinal residency: A review. *J. Biomed. Eng.* **3**, 042001 (2021).

16. B. H. Kim, K. Li, J.-T. Kim, Y. Park, H. Jang, X. Wang, Z. Xie, S. M. Won, H.-J. Yoon, G. Lee, W. J. Jang, K. H. Lee, T. S. Chung, Y. H. Jung, S. Y. Heo, Y. Lee, J. Kim, T. Cai, Y. Kim, P. Prasopsukh, Y. Yu, X. Yu, R. Avila, H. Luan, H. Song, F. Zhu, Y. Zhao, L. Chen, S. H. Han, J. Kim, S. J. Oh, H. Lee, C. H. Lee, Y. Huang, L. P. Chamorro, Y. Zhang, J. A. Rogers, Three-dimensional electronic microfliers inspired by wind-dispersed seeds. *Nature* **597**, 503–510 (2021).

17. G. Gu, J. Zou, R. Zhao, X. Zhao, X. Zhu, Soft wall-climbing robots. *Sci. Robot.* **3**, eaat2874 (2018).

18. S. D. de Rivaz, B. Goldberg, N. Doshi, K. Jayaram, J. Zhou, R. J. Wood, Inverted and vertical climbing of a quadrupedal microrobot using electroadhesion. *Sci. Robot.* **3**, eaau3038 (2018).

19. O. Unver, M. Sitti, Flat dry elastomer adhesives as attachment materials for climbing robots. *IEEE Trans. Robot.* **26**, 131–141 (2010).

20. M. P. Murphy, C. Kute, Y. Mengüç, M. Sitti, Waalbot II: Adhesion recovery and improved performance of a climbing robot using fibrillar adhesives. *Int. J. Rob. Res.* **30**, 118–133 (2011).

21. M. R. Cutkosky, Climbing with adhesion: From bioinspiration to biounderstanding. *Interface Focus* **5**, 20150015 (2015).

22. Y. Tang, Q. Zhang, G. Lin, J. Yin, Switchable adhesion actuator for amphibious climbing soft robot. *Soft Robot.* **5**, 592–600 (2018).

23. J. Huang, Y. Liu, Y. Yang, Z. Zhou, J. Mao, T. Wu, J. Liu, Q. Cai, C. Peng, Y. Xu, Electrically programmable adhesive hydrogels for climbing robots. *Sci. Robot.* **6**, eabe1858 (2021).

24. M. Boegh, H. M. Nielsen, Mucus as a barrier to drug delivery—Understanding and mimicking the barrier properties. *Basic Clin. Pharmacol. Toxicol.* **116**, 179–186 (2015).

25. R. Shaikh, T. R. R. Singh, M. J. Garland, A. D. Woolfson, R. F. Donnelly, Mucoadhesive drug delivery systems. *J. Pharm. Bioallied Sci.* **3**, 89–100 (2011).

26. J. Li, A. Celiz, J. Yang, Q. Yang, I. Wamala, W. Whyte, B. Seo, N. Vasilyev, J. Vlassak, Z. Suo, Tough adhesives for diverse wet surfaces. *Science* **357**, 378–381 (2017).

27. M. Sitti, R. S. Fearing, Synthetic gecko foot-hair micro/nano-structures as dry adhesives. *J. Adhes. Sci. Technol.* **17**, 1055–1073 (2003).

28. M. Murphy, B. Aksak, M. Sitti, Gecko-inspired directional and controllable adhesion. *Small* **5**, 170–175 (2009).

29. M. P. Murphy, S. Kim, M. Sitti, Enhanced adhesion by gecko-inspired hierarchical fibrillar adhesives. *ACS Appl. Mater. Interfaces* **1**, 849–855 (2009).

30. S. Gorb, M. Varenberg, A. Peressadko, J. Tuma, Biomimetic mushroom-shaped fibrillar adhesive microstructure. *J. R. Soc. Interface* **4**, 271–275 (2007).

31. D. Son, V. Liimatainen, M. Sitti, Machine learning-based and experimentally validated optimal adhesive fibril designs. *Small* **17**, 2102867 (2021).

32. S. Kim, M. Sitti, Biologically inspired polymer microfibers with spatulate tips as repeatable fibrillar adhesives, *Appl. Phys. Lett.* **89**, 261911 (2006).

33. B. Luk, D. Cooke, A. Collie, N. Hewer, S. Chen, Intelligent legged climbing service robot for remote inspection and maintenance in hazardous environments. in *Proceedings of 8th IEEE*

Conference on Mechatrinics and Machine Vision in Practice (2001), 17 to 29 August 2001, pp. 2342–2347.

34. S. T. Choi, Extended JKR theory on adhesive contact of a spherical tip onto a film on a substrate. *J. Mater. Res.* **27**, 113–120 (2012).

35. G. J. Tortora, S. R. Grabowski, The respiratory system & the digestive dystem, in *Principles of Anatomy and Physiology* (Wiley, 2003), pp. 850–952.

36. Y.-J. Lim, D. Deo, T. P. Singh, D. B. Jones, S. De, In situ measurement and modeling of biomechanical response of human cadaveric soft tissues for physics-based surgical simulation. *Surg. Endosc.* **23**, 1298–1307 (2009).

37. M. Eskandari, A. L. Arvayo, M. E. Levenston, Mechanical properties of the airway tree: Heterogeneous and anisotropic pseudoelastic and viscoelastic tissue responses. *J. Appl. Physiol.* **125**, 878–888 (2018).

38. J. Rains, J. Bert, C. Roberts, P. Pare, Mechanical properties of human tracheal cartilage. *J. Appl. Physiol.* **72**, 219–225 (1992).

39. C. Scholz, J. T. Engelder, The role of asperity indentation and ploughing in rock friction—I: Asperity creep and stick-slip. *Int. J. Rock Mech. Min.* **13**, 149–154 (1976).

40. H. Yuk, T. Zhang, S. Lin, G. A. Parada, X. Zhao, Tough bonding of hydrogels to diverse non-porous surfaces. *Nat. Mater.* **15**, 190–196 (2016).

41. H. Yuk, J. Wu, T. L. Sarrafian, X. Mao, C. E. Varela, E. T. Roche, L. G. Griffiths, C. S. Nabzdyk, X. Zhao, Rapid and coagulation-independent haemostatic sealing by a paste inspired by barnacle glue. *Nat. Biomed. Eng.* **5**, 1131–1142 (2021).

42. J. Yang, R. Bai, B. Chen, Z. Suo, Hydrogel adhesion: A supramolecular synergy of chemistry, topology, and mechanics. *Adv. Funct. Mater.* **30**, 1901693 (2020).

43. B. P. Lee, P. B. Messersmith, J. N. Israelachvili, J. H. Waite, Mussel-inspired adhesives and coatings. *Annu. Rev. Mater. Sci.* **41**, 99–132 (2011).

44. H. Lee, B. P. Lee, P. B. Messersmith, A reversible wet/dry adhesive inspired by mussels and geckos. *Nature* **448**, 338–341 (2007).

45. J. Zhang, Z. Ren, W. Hu, R. H. Soon, I. C. Yasa, Z. Liu, M. Sitti, Voxelated three-dimensional miniature magnetic soft machines via multimaterial heterogeneous assembly. *Sci. Robot.* **6**, eabf0112 (2021).

46. A. Abramson, M. R. Frederiksen, A. Vegge, B. Jensen, M. Poulsen, B. Mouridsen, M. O. Jespersen, R. K. Kirk, J. Windum, F. Hubálek, Oral delivery of systemic monoclonal antibodies, peptides and small molecules using gastric auto-injectors. *Nat. Biotechnol.* **40**, 103–109 (2022).

47. S. Yim, E. Gultepe, D. H. Gracias, M. Sitti, Biopsy using a magnetic capsule endoscope carrying, releasing, and retrieving untethered microgrippers. *IEEE. Trans. Biomed.* **61**, 513–521 (2013).

48. S. Hua, Advances in oral drug delivery for regional targeting in the gastrointestinal tract—Influence of physiological, pathophysiological and pharmaceutical factors. *Front. Pharmacol.* **11**, 524 (2020).

49. M. E. Johansson, H. Sjövall, G. C. Hansson, The gastrointestinal mucus system in health and disease. *Nat. Rev. Gastroenterol. Hepatol.* **10**, 352–361 (2013).

50. C. Gao, J. Ren, C. Zhao, W. Kong, Q. Dai, Q. Chen, C. Liu, R. Sun, Xylan-based temperature/pH sensitive hydrogels for drug controlled release. *Carbohydr. Polym.* **151**, 189–197 (2016).

51. D. C. Leslie, A. Waterhouse, J. B. Berthet, T. M. Valentin, A. L. Watters, A. Jain, P. Kim, B. D. Hatton, A. Nedder, K. Donovan, A bioinspired omniphobic surface coating on medical devices prevents thrombosis and biofouling. *Nat. Biotechnol.* **32**, 1134–1140 (2014).

52. K. D. Fine, C. A. Santa Ana, J. L. Porter, J. S. Fordtran, Effect of changing intestinal flow rate on a measurement of intestinal permeability. *Gastroenterology* **108**, 983–989 (1995).

53. C. Zhong, T. Gurry, A. A. Cheng, J. Downey, Z. Deng, C. M. Stultz, T. K. Lu, Strong underwater adhesives made by self-assembling multi-protein nanofibres. *Nat. Nanotechnol.* **9**, 858–866 (2014).

54. P. E. Dupont, B. J. Nelson, M. Goldfarb, B. Hannaford, A. Menciassi, M. K. O'Malley, N. Simaan, P. Valdastri, G.Z. Yang, A decade retrospective of medical robotics research from 2010 to 2020. *Sci. Robot.* **6**, eabi8017 (2021).

55. G. Ciuti, R. Caliò, D. Camboni, L. Neri, F. Bianchi, A. Arezzo, A. Koulaouzidis, S. Schostek, D. Stoyanov, C. Oddo, Frontiers of robotic endoscopic capsules: A review. *J. Micro-Bio Robot.* **11**, 1–18 (2016).

56. M. Sitti, *Mobile Microrobotics* (MIT Press, 2017).

57. A. Kotikian, C. McMahan, E. C. Davidson, J. M. Muhammad, R. D. Weeks, C. Daraio, J. A. Lewis, Untethered soft robotic matter with passive control of shape morphing and propulsion. *Sci. Robot.* **4**, eaax7044 (2019).

58. L. Hines, K. Petersen, G. Z. Lum, M. Sitti, Soft actuators for small-scale robotics. *Adv. Mater.* **29**, 1603483 (2017).

59. X. Ji, X. Liu, V. Cacucciolo, M. Imboden, Y. Civet, A. El Haitami, S. Cantin, Y. Perriard, H. Shea, An autonomous untethered fast soft robotic insect driven by low-voltage dielectric elastomer actuators. *Sci. Robot.* **4**, eaaz6451 (2019).

60. Q. Liu, W. Wang, M. F. Reynolds, M. C. Cao, M. Z. Miskin, T. A. Arias, D. A. Muller, P. L. McEuen, I. Cohen, Micrometer-sized electrically programmable shape-memory actuators for low-power microrobotics. *Sci. Robot.* **6**, eabe6663 (2021).

61. A. Hong, A.J. Petruska, A. Zemmar, B.J. Nelson, Magnetic control of a flexible needle in neurosurgery. *IEEE Tran. on Biomed. Eng.,* **68**, 616–627 (2021).

62. Z. Yang, L. Zhang, Magnetic actuation systems for miniature robots: A review. *Adv. Intell. Syst.* **2**, 2000082 (2020).

63. A. Tittel, E. Schippers, V. Grablowitz, M. Pollivoda, M. Anurov, A. Öttinger, V. Schumpelick, Intraabdominal humidity and electromyographic activity of the gastrointestinal tract. Laparoscopy versus laparotomy. *Surg. Endosc.* **9**, 786–790 (1995).
